# Temporal and Spatial Evolution of Brain Network Topology during the First Two Years of Life

**DOI:** 10.1371/journal.pone.0025278

**Published:** 2011-09-23

**Authors:** Wei Gao, John H. Gilmore, Kelly S. Giovanello, Jeffery Keith Smith, Dinggang Shen, Hongtu Zhu, Weili Lin

**Affiliations:** 1 Department of Radiology and Biomedical Research Imaging Center, University of North Carolina at Chapel Hill, Chapel Hill, North Carolina, United States of America; 2 Department of Psychiatry, University of North Carolina at Chapel Hill, Chapel Hill, North Carolina, United States of America; 3 Department of Psychology and Biomedical Research Imaging Center, University of North Carolina at Chapel Hill, Chapel Hill, North Carolina, United States of America; 4 Department of Radiology, University of North Carolina at Chapel Hill, Chapel Hill, North Carolina, United States of America; 5 Department of Biostatistics and Biomedical Research Imaging Center, University of North Carolina at Chapel Hill, Chapel Hill, North Carolina, United States of America; Queensland Institute of Medical Research, Australia

## Abstract

The mature brain features high wiring efficiency for information transfer. However, the emerging process of such an efficient topology remains elusive. With resting state functional MRI and a large cohort of normal pediatric subjects (n = 147) imaged during a critical time period of brain development, 3 wk- to 2 yr-old, the temporal and spatial evolution of brain network topology is revealed. The brain possesses the small world topology immediately after birth, followed by a remarkable improvement in whole brain wiring efficiency in 1 yr olds and becomes more stable in 2 yr olds. Regional developments of brain wiring efficiency and the evolution of functional hubs suggest differential development trend for primary and higher order cognitive functions during the first two years of life. Simulations of random errors and targeted attacks reveal an age-dependent improvement of resilience. The lower resilience to targeted attack observed in 3 wk old group is likely due to the fact that there are fewer well-established long-distance functional connections at this age whose elimination might have more profound implications in the overall efficiency of information transfer. Overall, our results offer new insights into the temporal and spatial evolution of brain topology during early brain development.

## Introduction

Consisting of billions of neurons and trillions of synapses, the human brain represents perhaps one of the most complex systems in the world. Despite the fact that grasping the organizational principle of such a complex system is incredibly appealing, our understanding of the emergence and organizational processes of human brain networks remains largely elusive. By designating different brain regions as nodes and connections between them as edges, promising results based on the graph theory have been demonstrated in modeling the complex brain networks [1], [2], [3], [4], [5], [6]. Specifically, the small-world topology [7] appears well suited to quantitatively characterize the complex network topology of the human brain. The small world topology implicates networks that exhibit densely connected local neighborhoods to achieve a higher cliquishness than a random network, while concurrently exerting direct long-range connections to distant regions to achieve a shorter path length than a regular network, leading to excellent local and global wiring efficiency for information transfer. An additional feature of this topology is the presence of highly important brain regions (hubs) that bridge disparate and local clusters to achieve a high global efficiency [8]. Such nodes most likely act as important portals controlling information transfer within the system. However, unlike the scale-free network where impairments/attacks targeting hubs lead to profound compromise of brain wiring efficiency, the small world topology possesses high resilience to both random errors and attacks to the hubs [3].

Extensive studies have reported that a mature human brain exhibits a small world topology [1], [3], [7], [9]. Furthermore, Fransson et al [10] showed that the infant (1 week old) brain network also exhibits a small world topology. However, how the brain network topology of a newborn continues to evolve toward its mature form remains poorly understood, especially during the first years of life. In particular, the elongation of major white matter tracts is not completed until approximately 9 months of age while the myelination process will continue into late childhood or even adolescence [11], [12], [13]. Such combined maturation process has profound implications on achieving a highly efficient brain network as well as its resilience to targeted attacks. Therefore, despite the observed small world topology in newborns, substantial evolution of brain network topology during early brain development is highly anticipated. In this study, we aim to shed new light on the temporal and spatial evolution of brain network topology during the first two years of normal brain development.

From the perspective of early brain network developments, several major physiological parameters will most likely concurrently evolve as a function of age, including, potentially, brain functional regions (nodes), number of connections among different brain regions (edges), connection strengths and parameters associated with network topologies (degree, efficiency[14], small-worldness[7], and so on). As a result, it is unlikely to address all of these variables in a single study. In the context of our study, we specifically focus on the evolution of brain functional network topologies during the first two years of life. Therefore, to minimize the number of variables, we have chosen to keep the number of brain functional regions as well as the number of connections (cost) among the brain regions constant so as to allowing a direct assessment of how the re-organization of a fixed number of connections among brain regions leads to changes of small world properties. We hypothesize that there will be significant improvements in small world properties, particularly wiring efficiencies from 3 wks to 1 yr of age given the establishment of long-range axonal connections during this time period and it will be more stable after 1 yr of age. With less matured long-distance connections at birth when compared to that in 1 and 2 yr olds, we further hypothesize that the brain wiring topology in neonates will be largely locally connected but evolving to a globally distributed network after 1 yr of age thanks to the continuing maturation of long distance connections. Finally, such under-developed long-distance connections might make nodes that possess direct connections with distant brain regions much more important for the wellbeing of the whole network than other nodes, which in turn will make the neonatal brain network more susceptible to impairments/attacks targeting at these hubs.

In this study, resting state functional connectivity MRI was conducted on a large cohort (n = 147) of naturally sleeping healthy normal pediatric subjects, including 51 neonates (27 male, 23±12 days (SD)), 50 1 yr olds (27 male, 13±1 months) and 46 2 yr olds (28 male, 24±1 months) (Text S1, supporting information (SI)). Quantitative analyses of whole brain and regional small world properties were first evaluated, followed by investigating brain hubs and finally, determining resilience of the brain to random errors and targeted attacks from 3 wks to 2 yrs of age.

## Results

**Figure 1:**
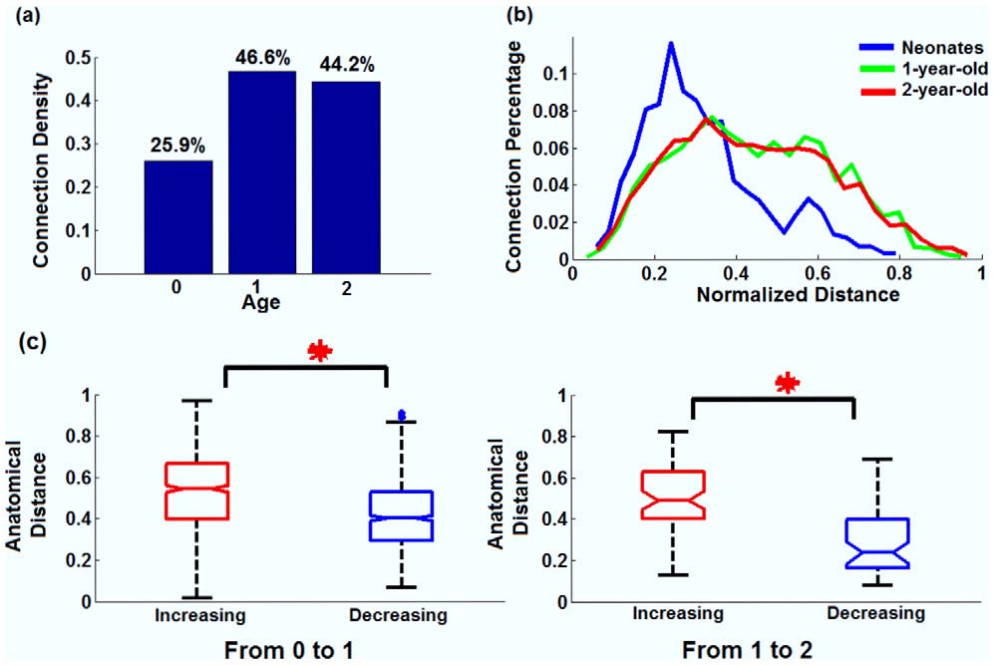
Age-dependent growth pattern of functional connections. (a) the connection density plot of all three age groups (based on statistical testing of connections (P<0.05, FDR corrected)); (b) the histogram plots of the normalized anatomical distance associated with the significant connections for each age group; (c) statistical comparisons of the anatomical distances associated with those connections that exhibit significantly changed strength with age. Red: increasing connections with age; blue: decreasing connections with age.

The brain was parcellated into ninety regions-of-interest (ROIs)/nodes encompassing both cortical and subcortical areas [15] (Details in Tables S1 and S2). Functional connectivity between each pair of ROIs was defined as the magnitude of Pearson correlation of the low frequency (<0.08 Hz) spontaneous blood oxygen level dependent (BOLD) fluctuations. Subsequently, individual correlation matrices (90×90) were Fisher z-transformed and averaged to obtain a group mean. Two-way t-test was conducted to determine statistical significance of a given connection. Although our study largely focuses on how brain network topology evolves during early brain development when the number of connections is kept as a constant across different age groups, given the anticipated changes of number of connections during normal brain development, Fig. 1 shows the alteration of connection density when the number of edges is not kept as a constant. As one would have expected, a major increase in the connection density from neonates to 1 yr olds (from 25.9% to 46.6%) is observed (Fig. 1a). Interestingly, however, the connection density is relatively stable from 1 to 2 yrs (44.2%) of age. The histograms of anatomical distances associated with the significant connections show a remarkable right-ward shift from neonates to 1 yr and 2 yr olds, indicating the emergence of long-distance connections from neonates to 1 yr of age (Fig. 1b). To more explicitly explore the relationship between the changes of connection strength (either decrease or increase) and the anatomical distance associated with a given connection, statistical comparisons were performed. In total, 588 connections (14.52%) show significantly increased connectivity strength while 818 (20.2%) exhibit significantly decreased connectivity strength from neonates to 1 yr olds. In contrast, 63 (1.6%) and 49 (1.2%) connections demonstrate increased and decreased strength from 1 to 2 yrs of age, respectively, indicating again the non-linear developmental pattern. More importantly, as shown in Fig. 1c, the connections exhibiting an increased strength consistently possess significantly longer anatomical distances than those with decreased strength (p = 1.32e-23 for 0 to 1 and p = 1.25e-6 for 1 to 2 yrs of age), strongly supporting the notion of the growth of long-distance connections during this period of time. The anatomical distribution of these significantly changing connections is provided in Fig. S1.

**Figure 2:**
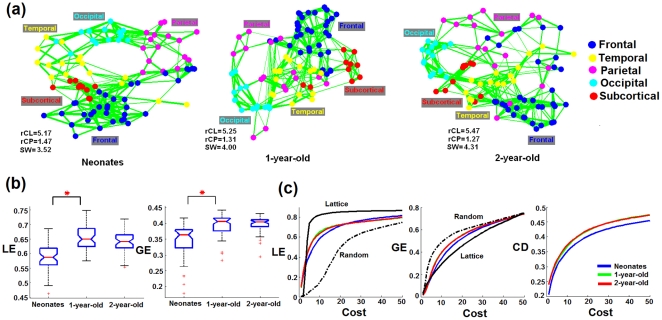
The evolution of brain's topology and small-world properties. (a) The group mean correlation matrices at a cost of 10% are visualized using spring embedding plots for all three groups. Nodes are color coded with respect to the lobe they belong to. Each edge represents the mean connectivity strength between a pair of nodes. The *rCL* and *rCP* represent 
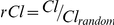
and 
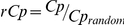
where *CL* and *CP* are the clustering coefficient and characteristic path length for a given subgraph and the subscript “random” indicates a random network. SW represents the small-worldness measure; (b) Statistical comparison of LE and GE at cost 10% based on individual subject's correlation matrices. Red asterisks represents significant difference at p<0.05 (FDR correction); (c) LE, GE, and CD curve across the cost range of 1% to 50%. Significant increase of LE occurs from neonates to 1 yr olds for a range of cost spanning from 2% through 21% and GE from 4% to 44% (p<0.05, FDR corrected). The calculation was based on individual subjects and the mean values for each age group are plotted.

As developmental changes in connection density/distance are clearly demonstrated in Fig. 1, we sought to further delineate the age-dependent evolution of functional topologies. In this examination, a fixed cost (the number of connections over all possible ones) for different age groups was employed. In so doing, the observed age-dependent differences in functional topology as well as efficiency measures should only reflect the re-organization of the connections but not different number of connections. P-values were used as threshold to generate sparse correlation matrices at different costs. The group mean correlation matrices at a cost of 10% (top 10% of the most strongly connected regions based on their corresponding p-values) were visualized using spring embedding plots for all three groups (Fig. 2a), which places strongly connected nodes geometrically closer and vice versa. As shown, with the exception of few regions in the frontal and temporal lobes, the functional topology of 3 wk old neonates shows that the brain regions within each lobe are highly connected (geometrically close with each other in the spring embedding plots). In addition, the network topology also preserves the anatomical relation of different lobes (e.g., the parietal lobe is between the frontal and occipital lobes). While the network topology remains reasonably organized in 1 yr and 2 yr olds, more long-range between-lobe connections (less within lobe connections) are observed especially for the parietal lobe in 1 yr olds and the parietal/frontal lobes in 2 yr olds. These dynamic changes of network topologies are consistent with the dramatic growth of long-distance connections as shown in Fig. 1. Such topological changes are also reflected in the measurements of small-worldness (SW); the brain topology of all age groups exhibits the small-world topology with an age-dependent increase in SW (SW  =  3.52 in neonates, 4.00 in 1 yr olds, and 4.31 in 2 yr olds).

To further determine brain wiring efficiency, local (LE) and global efficiency (GE) for the whole network were calculated in each individual subject and the results are presented in Fig. 2b. Significant increases of LE and GE are observed from 3 wks to 1 yr of age (red asterisks, p = 2e-9 and 1e-8 for LE and GE, respectively) but neither measure is statistically different between 1 and 2 yrs of age. To role out the potential bias for choosing a specific fixed threshold (10% of the cost) for the above analysis, similar analyses were conducted through a series of costs ranging from 1% to 50% with a step size of 1% and the results are presented in Fig. 2c. As shown, the LE and GE curves of all three age groups lie in between those of the random and regular network throughout most of the cost range, supporting the existence of small-world property for all three age groups. Statistical analysis reveals significant increase of LE from neonates to 1 yr olds for a range of costs spanning from 2% through 21% and GE from 4% to 44% (p<0.05, FDR corrected). Again, no statistical difference between 1 yr olds and 2 yr olds is observed for either LE or GE at any costs.

As consistently shown in Fig. 1 and Fig. 2a, the most dramatic topological change in 1 yr and 2 yr olds comparing with neonates is the increased number of long-distance between-lobe connections. The fact that this topological change coincides with the significant increase of global efficiency is expected since it is most likely that the establishment of long-range connections (between lobes) connects different local clusters and improves the global efficiency. Moreover, the measurements of mean connection distance (CD) among the connected regions within functional networks constructed across the cost range of 1%∼50% are provided in Fig. 1c. Strikingly, the GE and CD curves across all costs are highly similar in shape, which strongly supports the notion that the development of long-distance connections may play a critical role for the improvements of global efficiency. While results in Fig. 2 were obtained by fixing the cost across different age groups, additional results obtained by fixing the p-value/connection strength threshold while allowing the cost to vary are provided in Fig. S2a/Fig. S3a for thresholded connectivity matrices, Fig. S2b/Fig. S3b for the spring-embedding plots, and Fig. S2c/Fig. S3e for the comparison of LE and GE between different age groups, respectively (supplementary materials).

**Figure 3:**
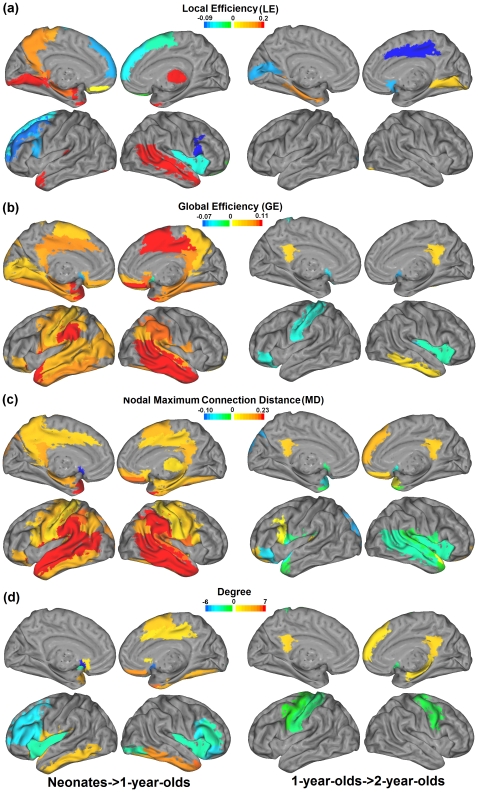
Regional developments of LE (a), GE (b), nodal maximum connection distance (MD) (c) and Degree (d). Both regional increase (represented by yellow to red colors) and decrease (represented by green to blue colors) are shown on the same brain surface. The left column shows the changes from neonates to 1 yr olds while the right column shows the changes from 1 yr to 2 yr olds.

While the above global measurements are informative, regional comparisons of LE, GE, nodal maximum connection distance (MD, defined as the normalized longest connection distance exerted from a given region), and degree (number of connections that a particular node makes) were also conducted to provide further insights into the temporal and spatial evolution of brain topology. All regional measures were calculated for costs ranging between 4% and 21% which were then averaged for each parameter, separately, for subsequent statistical comparisons. This choice of cost range stems from the following two reasons: (1) this range is well within the small-world regime (see Fig. 2) and is consistent with other studies reported in the literature [2], [3]; and (2) significant age-dependent increases of both LE and GE are observed within this range (Fig. 2c). The surface rendering of significant developmental changes (p<0.05, uncorrected) are shown in Fig. 3 and the detailed lists of involved regions are provided in Table. S3, S4, S5, S6 for LE, GE, MD, and degree, respectively. Significant regional changes detected based on two single costs (10% and 15%) are also presented in Fig. S4 and Fig. S5, including LE (Fig. S4a and S5a), GE (Fig. S4b and S5b), MD (Fig. S4c and S5c) and degree (Fig S4d and S5d), which show high consistency with that in Fig. 3. In addition, the parameter maps of each measure in different age groups are presented in Fig. S6 (LE), Fig. S7 (GE), Fig. S8, (MD) and Fig. S9 (degree), respectively.

As shown in Fig. 3, generally more extensive regional developments occur from neonates to 1 yr olds than from 1 yr olds to 2 yr olds, which is consistent with the temporal trend shown in Fig. 1, 2. However, new insights emerge from the regional development patterns. For LE, while Fig. 2b, c only show a global increase during the first year, both regional increase and decrease are observed in Fig. 3a indicating the advantage of regional examinations in delineating local differential growth patterns. Specifically, the regional increases are observed in temporal, occipital, as well as several subcortical regions while the regional decreases are generally focused on frontal regions (especially lateral frontal, Table S3). For GE (Fig. 3b) and MD (Fig. 3c), their spatial development patterns during the first year are highly consistent; extensive regional enhancement are observed with most significant changes in temporal and parietal lobe while regional decreases only occur in three subcortical regions (bilateral Caudate and left Putamen) for both parameters. The high consistency between these two parameters reinforces the importance of long-distance connections in establishing global efficiency. Finally, for degree (Fig. 3d), again both regional increases and decreases are observed and more interestingly a large portion of the significant degree changes overlaps with corresponding changes in LE (highlighted in green in Table. S3 and S6), which potentially suggests a significant role of degree in nodal wiring efficiency.

During the second year, the changes are much sparser but more focused and important trend emerges by considering the regional changes of different parameters simultaneously. Specifically, we observe that during this time the regional increase for GE, MD, and degree are highly consistent (highlighted in red in Table S4, S5, S6) and concentrates on bilateral posterior cingulate cortex (GE, MD, and degree) and right superior medial frontal cortex (MD and degree), which are consistently reported as two major hubs in the so called “default-mode” network [16], [17], [18]. Moreover, the other regions showing significant increase of either one of these three parameters include right inferior temporal lobule (GE), right superior orbital frontal cortex/left middle orbital frontal cortex (MD), and right hippocampus (degree), which are also included in or related to the “default-mode” network [16], [17]. Such a converging enhancement may indicate a dramatic development of this higher order cognitive network during the second year of life. In great contrast, for both GE and degree, most of the regional decreases are observed in the pre-/post-central cortex as well as caudate regions (highlighted in blue in Table S4, S6), which are apparently primary-function related. For MD, similar pattern exists (highlighted in blue in Table S5) though substantial regional decreases in other parts of the brain are also observed. Overall, such an interesting increasing/decreasing spatial trend indicates differential development patterns for primary and higher order cognitive functions during the second year of life.

**Figure 4:**
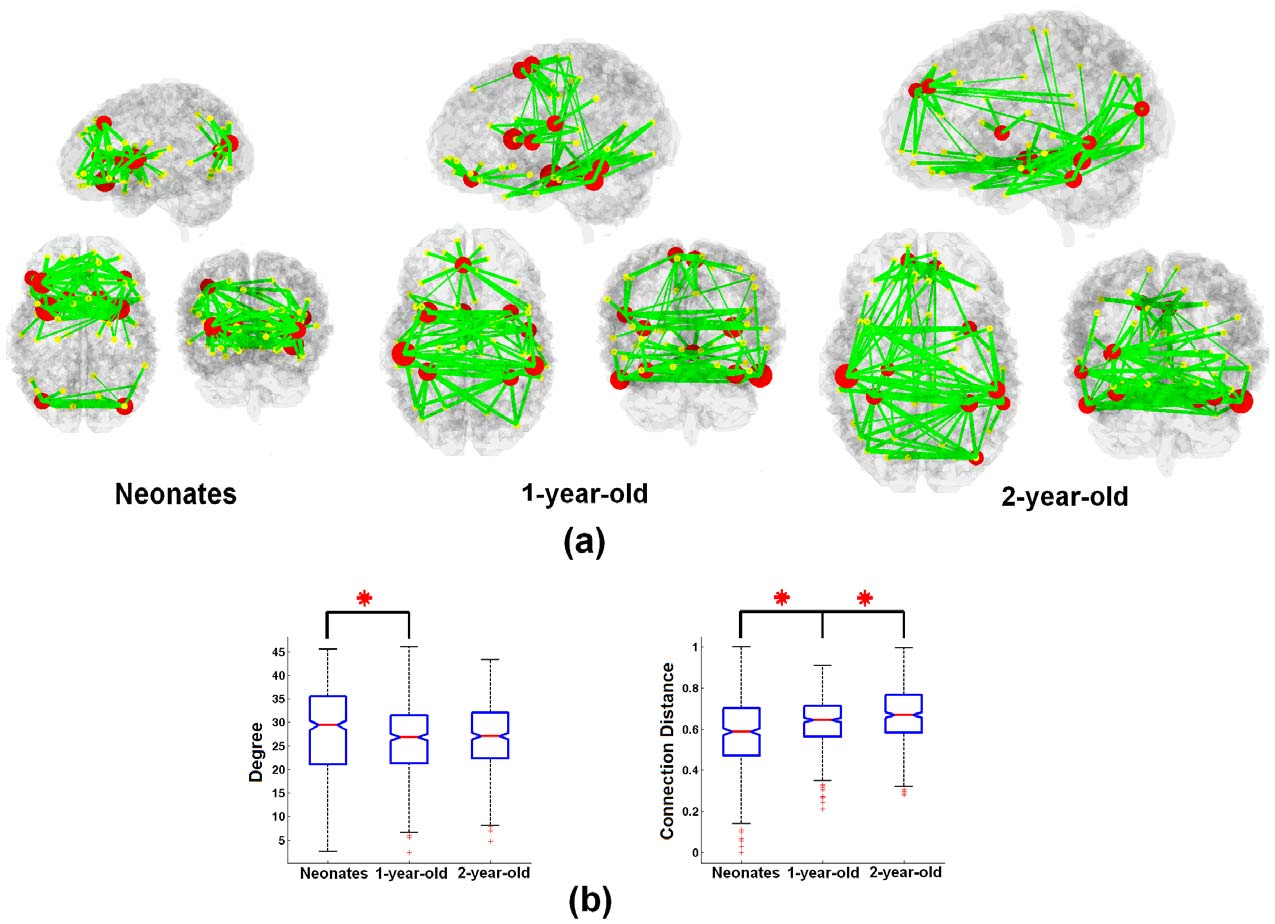
The temporal and spatial evolution of brain hubs during the first two years of life. (a) Spatial distribution of the top 10 hubs and their associated connections at cost 10% (based on the group-mean connectivity matrix) is shown; (b) The degree and maximum connection distance (MD) of the top 10 hubs are compared across different age groups. Red asterisks represents significant difference at p<0.05 (FDR correction).

To further delineate the potential functional hubs during this early period and examine their temporal evolution, the betweenness centrality (BC) [19] was calculated for each age group. For visualization purposes, the brain regions with the top 10 BC values in each age group are shown in Fig. 4 while the full lists of all regional BC values are presented in Fig. S10. Substantial temporal evolution of hubs/connections across age groups is apparent (Fig. 4a). There are two apparent clusters of hubs, anterior and posterior clusters, in neonates, suggesting that major functional developments may have already occurred in both the frontal and occipital lobes. In addition, most of the connections between hubs are interhemispherical with no clear connections between the two clusters. In 1 yr olds, the hubs are well connected and more anatomically centered with few hubs located in the anterior and posterior areas of the brain as that seen in neonates. Finally, the distribution of hubs becomes more spatially uniform in 2yr olds. The hubs in neonates collectively possess a higher degree than 1 yr olds (red asterisks, p = 5.00e-5) while 1 and 2 yr olds are comparable (Fig. 4b). However, significant increase of MD appears to persist from neonates to 2 yrs of age (p = 1.06e-7, 8.87e-6 between neonates/1 yr and 1 yr/2 yrs, respectively). Such a pattern suggests that the hubs possess an increasingly more efficient strategy – less but longer connections to achieve a higher global efficiency (Fig. 2).

For spatial distribution of hubs (Fig. 4, Fig. S10), clearly there is a major focus on primary functions during early infancy: For motor-related function, bilateral caudate (known to be associated with voluntary movement control [20], [21]) emerge as hubs in neonates; bilateral SMA are among the hubs in 1 yr olds; For visual functions, bilateral Occpt-M appear in neonates; bilateral Temp-I and Fusiform regions are among the hubs in both 1 and 2 yr olds; besides, left Occpt-M also emerges in 2 yr olds. Such a primary function emphasis is consistent with the findings reported by Fransson et al [10], which indicates again the importance of primary functions during early development. However, the emergence of bilateral Frt-S-M regions in two year olds is consistent with the regional development patterns shown in Fig. 3, which may indicate a gradual shift of developmental focus to higher order cognitive functions during this period of time, especially those related with the default network. Finally, another interesting finding is the bilateral insula areas are consistently observed as hubs for all three age groups (Fig. S10).

**Figure 5:**
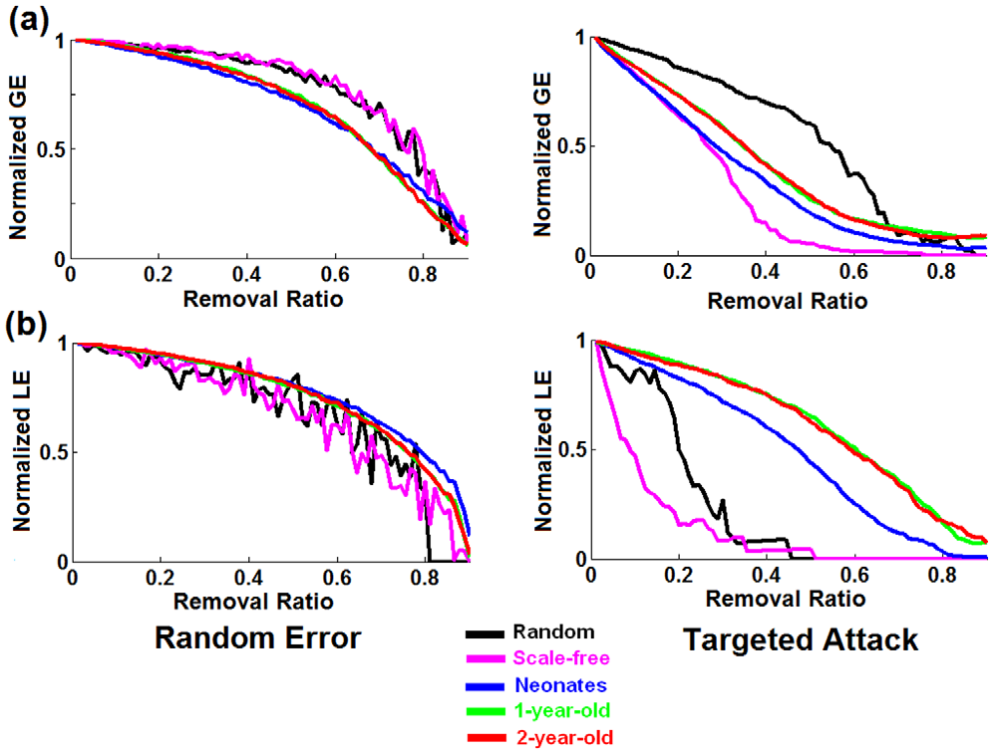
Brain's resilience to random errors and targeted attacks in comparison with random and scale free networks at cost 10%. (a) Effects of random errors (left) and targeted attacks (right) on GE; (b) Effects of random errors (left) and targeted attacks (right) on LE. All efficiency values were normalized to the corresponding values of an intact network.

Finally, given the small world topology and existence of hubs, it is imperative to determine how resilient the brain is for sustaining both random errors and targeted attacks in this critical time period of brain development. Specifically, random errors were simulated by randomly eliminating one node together with all its connections. Both LE and GE were subsequently recalculated. In targeted attacks, the node removal followed a descending order based on the BC measures. This process was repeated until 90% of all nodes were eliminated for both random errors and targeted attacks. Results obtained from our study cohorts were then compared with both random and scale-free networks [8]. The resilience results measured by both GE and LE at cost of 10% are presented in Fig. 5 (other costs in Fig. S11, and S12 for GE and LE, respectively). For GE, all three age groups demonstrate relatively high resilience to random errors, although they are slightly lower than the random and scale-free networks (Fig. 5a, left column). In contrast, all three groups outperform the scale-free network (Fig. 5a, right column) in sustaining targeted attacks, consistent with the reported results in the literature [3], [8], [22]. Nevertheless, at a removal ratio < 0.3, the normalized GE of neonates is identical to that of a scale free network under targeted attacks. For LE, all three age groups show similar resilience against random errors as that of the random and scale-free networks but much higher resilience against targeted attacks (Fig. 5b). More importantly, clear age-related improvements in resilience, particularly against targeted attacks are observed for both LE and GE (Fig. 5). Again, the improvement is only significant during the first year of life (p = 6.41e-8/1.35e-4 for LE/GE, respectively), which is consistent with the observed developmental pattern of brain efficiency. Area under curve (AUC) of similar curves as that shown in Fig. 5 but across the cost range of 1%–50% was calculated to examine brain's resilience against random errors and targeted attacks at different costs. For GE, a significant increase of resilience to random errors is detected from neonates to 1 yr olds between costs 12% and 50% while no difference is detected between 1 and 2 yr olds (p<0.05, FDR corrected). In addition, a significant increase of resilience is detected from neonates to 1 yr olds between cost 4% and 50% for targeted attacks and similarly no difference is detected between 1 and 2 yr olds (p<0.05, FDR corrected). For LE, a significant increase of resilience to targeted attacks is detected from neonates to 1 yr olds between costs 2% and 50% and no significant changes are observed from 1 to 2 yr olds (p<0.05, FDR corrected). Finally, a comparison of resilience to the simulated attacks between our study cohort and synthetic small-world networks based on the Watts&Strogatz's model [7] is presented in Fig. S13. Briefly, the resilience curves of GE against random errors and LE against both random errors and targeted attacks from the pediatric groups lie either between or close to the curves of the synthetic networks (with different rewiring probabilities from 0.1 to 1). However, the synthetic small-world networks clearly show higher resilience for GE against targeted attacks than the real pediatric networks. This is likely due to the fact that the synthetic networks are rewired from lattice networks where all nodes have the same degree and each connection has the same probability to be rewired. As a result, all nodes are approximately equally important resulting in higher resilience to targeted attacks.

## Discussion

The temporal and spatial development processes of the brain network topology during a critical time period of brain development – from 3 wks to 2 yrs of age were revealed. Measurements of local efficiency, global efficiency, degree, anatomical connection distances, betweenness centrality and brain resilience to random errors and targeted attacks were provided. Although the brain networks for all three age groups exhibit small-world property based on the small-worldness measure, significant and non-linear increases of both LE and GE during the first two years of life are observed. Regional analysis further demonstrates differential developmental pattern between primary functions and higher order cognitive functions, especially during the second year of life. Finally, the evolution of network topology and hubs make the brain more resilient to both random errors and targeted attacks as age grows.

### Temporal and spatial evolution of brain network topology

Axons undergo a period of rapid elongation and establish extensive synapses/connectivity to their intra- and subcortical targets from midgestation through infancy [23]. These dramatic developments of structural connectivity appear to set the foundation for the concurrent optimization of brain wiring efficacy before birth [24], [25], [26], which is consistent with the observed small world topology in neonates in this study (Fig. 2a and 2c). Moreover, significant improvements of both LE and GE are observed from neonates to 1 yr olds whereas no statistical difference between 1 and 2 yr olds is observed (Fig. 2b and 2c). This non-linear developing pattern of brain wiring efficiency, particularly GE, is intriguing. Neonates demonstrate an anatomically ordered architecture (Fig. 2a), in which neighboring clusters are largely sequentially connected, leading to a longer path length (1.47, Fig. 2a), while 1 and 2 yr olds show considerably better wired brain topologies with abundant bridging connections even between distant clusters (decreased mean path length and increased GE), suggesting that anatomical long-range connections play a critical role in the observed improvement. Indeed, quantitative comparisons of connectivity strength show remarkable growth of long-distance connections (Fig. 1) and measures of the mean connection distance across all costs are surprisingly similar to that of GE (Fig. 2c), strongly supporting the anatomical basis of network efficiency and suggesting that the establishment of a higher GE rests critically on the development of long-distance connections. Increase in long-distance connections as a function of age has been reported when comparing school-aged children with adults [5], [11], [27]. Nevertheless, given the age differences between our study cohort and that reported in the literature, the biological underpinnings associated with the observed increased long-range connections most likely differ. Specifically, since the axonal properties (e.g., length, diameter, density) are most likely well established in school-aged children, the observed increase in long-range connectivity strength might primarily arise from the continuing myelination process, improving information transfer efficiency between distant brain regions [12]. In contrast, although some of the major long-range axonal connections especially those callosal fibers are present in neonates [12], [28], continuing axonal elongation/thickening is expected at this age to facilitate forming connections with other distant brain functional areas (e.g., in the anterior-posterior direction), especially during the first year of life [12]. Moreover, myelination process is also documented to experience the most dramatic growth during the first two years of life [12], [23], [29]. Therefore, the combined axonal growth and myelination process most likely account for the observed changes in our study.

The observation of no significant changes between 1 yr and 2 yr olds in whole brain small-world properties echoes the findings by Supekar et al [6] and Fair et al[5], who also failed to detect significant whole brain efficiency changes when comparing school-aged children with adults. However, significant regional wiring changes (LE/GE) have been documented in both previous studies [5], [6] as well as in the current one, which will be discussed in detail later. Combining these findings, it is plausible that the whole brain reaches an adult-like “small-world” topology from 1-year-old on while after that although significant local changes continues to reshape the brain for the development of specific functions, the whole brain efficiency property remains un-disturbed. However, the complete proof of this hypothesis needs further studies sampling the period between 2 yr olds studied here and school aged children studied by Supekar et al [6] and Fair et al. [5].

Additional insights into both temporal and spatial evolution of brain topologies during the critical time period of brain development emerge by further considering regional changes of small world properties. During the first year, extensive and convergent regional increase of GE and MD is observed, consistent with that observed in Fig. 2, as well as our hypothesis regarding the role of long distance connections in improving brain wiring efficiency. In contrast, both regional increases and decreases of LE are observed during this period, with the majority of regional changes accompanied by a concurrent change of degree (Fig. 3 and highlighted in green in Tables S3 and S6). Furthermore, regional differences are observed within the frontal and temporal lobes, separately, which warrant additional discussion. In the frontal lobe, remarkable reductions of LE (Fig. 3a and Table S3) and degree (Fig. 3d and Table S6) are observed among the lateral frontal regions (except for the right superior medial frontal and bilateral supplementary motor areas) whereas significant increases of GE are seen in the medial orbital frontal regions (Fig. 3b and Table S4). As the reduction of LE and degree may suggest regional specialization, potentially through the removal of redundant connections [12], our findings of concurrent reduction of LE and degree in the lateral frontal regions agree well with the reported evidence showing the initial development of various executive functions including working memory and joint attention during this time [30], [31]. In contrast, the increased GE in the orbital frontal regions might suggest the formation of the emotion processing network by exerting possible long-distance connections to the hippocampus and amygdala [32], which has been implicated for establishing attachment immediately after birth in juvenile monkeys [33].

Contrasting to the frontal lobe, which exhibits regional dissociation between decreased LE and increased GE, the entire temporal lobe demonstrates more uniform increase of all small world properties. Specifically, the temporal pole, including the left superior and bilateral mid temporal poles, shows a concurrent increase of LE, GE, and degree (Fig. 3 and Tables S3, S4, and S6). In contrast, the left Heschl gyrus and right mid temporal gyrus exhibit an increase of GE and LE, while the left inferior temporal gyrus shows an increase of GE and degree. Finally, the left mid temporal gyrus, bilateral superior temporal gyri, bilateral hippocampi, and bilateral parahippocampi show only an increase of GE. Together, these findings suggest that the temporal pole not only forms local connections, but also establishes long distance connections during this period of time.

Compared to the first year, alterations of small world properties during the second year are less extensive. However, our results suggest differential developmental trend for basic sensory and higher order functions. Specifically, the pre-/post-central gyri (motor-sensory) and caudate (autonomic control functions) [34], [35] demonstrate regional decreases of GE, MD and degree (highlighted in blue in Tables S4, S5, S6) during the first two year period. In contrast, the posterior cingulate and superior medial frontal regions (highlighted in red in Tables S4, S5, S6) exhibit a concurrent increase of GE, MD and degree, indicating dramatic development of the default network in the second year of life [16], [18], [36]. Given the widely reported function of this network including self-related thinking, mentalizing, planning, etc [16], [17], [37], these observations suggest more developmental focus for the higher-order cognitive functions in year 2, consistent with the functional network development reported in the literature [12], [38], [39], [40]. Particularly, the observed emphasis on default network echoes nicely with the emergence of self-consciousness during the second year of life as reported by Amsterdam [41]. Finally, these observations underscore the importance of a combined investigation of different measures and the need of regional analysis to further delineate locally specific development.

### Temporal and spatial evolution of brain hubs and their resilience to attacks

The spatial distribution of the hubs in early infancy clearly deviates from that reported in adults, which mainly reside in the higher-order cognitive foci, including prefrontal and medial parietal regions [24], [42]. Instead, the hubs in our study cohorts are more associated with motor and visual functions (Fig. 4, Fig. S10), consistent with the findings reported by Fransson et al [10]. These findings indicate early emphasis on primary functions during infancy period.

One of the interesting findings in the temporal developments of hubs is how consistent the insula is observed as a major hub for all three age groups (Fig. S10). This finding is intriguing since the insula is typically associated with higher-order functions. However, it has long been observed that the insula is the first cortex to differentiate and develop in fetus beginning from 6 weeks after conception [43], [44], providing the structural basis for its hub role immediately after birth. Moreover, the insula cortex is versatile and has diverging functions spanning from motor control, auditory processing, speech production, autonomic functions, to higher order decision making [44]. One potential explanation of our finding is that the functional role of insula also evolves with age; it governs primary functions like motor control and auditory processing during early infancy, but its role gradually evolves into the higher order functional domains through the experience-dependent learning process.

Given the small world topology and existence of hubs, it is imperative to determine how resilient the brain is for sustaining both random errors and targeted attacks in this critical time period of brain development (Fig. 5). For GE, all three age groups demonstrate excellent resilience to random errors similar to that of the random and scale-free networks while the ability to sustain targeted attacks outperforms the scale-free network but is inferior to the random network (Fig. 5a), which is consistent with the reported results in [3], [8], [22]. For LE, all three age groups show higher resilience to both random errors and targeted attacks than that of random and scale-free networks (Fig. 5b) although the differences are less apparent with higher costs (e.g., >40%, Fig. S12), indicating a better local organization of small-world topology in infants. Moreover, although the improved resilience to random errors is minimal, a clear age-related improvement in the resilience to targeted attacks is observed (Fig. 5), particularly between 3 wk and 1 yr olds, which is likely resulted from the spatial evolution of hubs and the established long-distance connections in 1 and 2 yr olds. Moreover, considering that the hubs are separated into anterior and posterior clusters in 3 wk olds (Fig. 4) and there are much fewer long-distance connections (Fig. 1, 2), one would suspect that this spatial pattern could lead to profound consequences on the brain's resilience, particularly under targeted attacks. Indeed, the reduction of GE in response to targeted attacks in neonates appears highly similar to the scale-free topology, particularly for a removal ratio less than 30% (Fig. S11). Collectively, these findings suggest that the neonatal brain is cliquishly connected so as to better preserving the LE while attack to the hubs is likely to result in substantial reduction in GE. In addition, the fact that the hubs in neonates collectively possess a higher degree than 1 yr and 2 yr olds (Fig. 4) is likely another factor leading to the observed lower resilience to targeted attacks (GE). Through considerable reorganization (particularly the development of long distance connections), the pediatric brain evolves to become more resilient to both random and targeted attacks.

### Limitations

As extensively studied in Van Wijk et al [45], there is currently no reliable and fully unbiased method to compare different empirical networks with varying number of nodes (N) and/or number of connections (K) using graph-theory based analysis. In the context of this study, N is the same across different age groups, but either fixing threshold allowing K to vary or vice versa has its own advantages and pitfalls. Most of the comparisons between different age groups reported in this study are based on fixing the cost (number of connections). While this approach facilitates statistical comparisons between different graphs, which is often practiced for graph theoretical analyses [2], [6], it is highly anticipated that the number of connections will changes during early brain development (see Fig. 1). With a fixed cost, however, our results will not reflect this biological alteration but rather reveal how brain topologies/efficiency are altered through rearrangement of connections. As a result, our results should be interpreted accordingly. For example, the regional changes shown in Fig. 3 might arise from a redistribution of the corresponding measures (e.g., degree) across ages rather than from absolute value changes given the imposed fixed cost threshold. Nevertheless, for the purpose of comparisons, additional results are shown in the supporting materials by fixing the threshold using either p-value (p<0.05, FDR corrected, Fig. S2) or connection strength (r>0.2, Fig. S3) and allowing the cost to vary across age groups. As shown in Fig. S2 and S3, the results obtained by applying these two different threholding methods are highly consistent with that using a fixed cost and demonstrate interesting patterns. First, the spatial topology pattern (Fig. S2b and Fig. S3b) remains qualitatively similar with that shown in Fig. 2 where the neonate networks are highly organized according to their anatomical lobe divisions while more between-lobe connections emerge in 1yr and 2 yr olds. Second, the anatomical histogram plot in Fig. S3d shows again an increased percentage of long-distance connections (similar to that in Fig. 1b). Finally, the global efficiency (GE) measure shows the similar age-dependent improvements as that in Fig. 2 although the local efficiency (LE) measure does not show significant changes across the first two years of life (Fig. S2c and Fig. S3e), partially supporting the robustness of the reported results against different thresholding methods (especially GE). Together, these results suggest that although similarity exists among the approaches of using different threshold, they also reflect the potential bias of varying connection density (K) on efficiency measurements.

Another potential limitation of the current study is the lack of monitoring of sleep stages, which could have effects on the functional connectivity strength. Future studies incorporating such monitoring (e.g., through simultaneous EEG recording) should be beneficial to minimize experimental variability.

### Conclusion

This study reveals temporal and spatial evolution of brain network topology from 3 wks to 2 yrs of age in a large cohort of normal and healthy subjects. To our knowledge, our study represents the first large scale study on normal brain network development during this critical time period of brain development. Three major findings are reported. First, immediately after birth, the brain already exhibits a small-world topology, implying a highly efficient brain network for information transfer. Continuing brain development, particularly the establishment of long-distance connections, leads to significant improvements for all small world properties from 3 wks to 1 yr of age. However, after 1 yr of age, the improvements in brain topology appear more regional than global, suggesting potential functional specialization. Specifically, the basic brain functions essential for subsequent development of higher order brain functions mature faster in infancy. However, the higher order brain functions gradually gain more emphasis during development. Second, the functional hubs are located in two well independent clusters, anterior and posterior clusters in neonates, but progressively move to centralized anatomical regions in 1 yr olds, and become more evenly distributed throughout the entire brain in 2 yr olds. Finally, the observed increase in long-distance connections and the spatial evolution pattern of functional hubs likely explains the age-dependent improvement of brain's resilience to both random errors and targeted attacks. Through the understanding of the normal temporal and spatial development of brain network topology, our findings could potentially pave the way for future delineation of brain disorders with neurodevelopmental origins.

## Materials and Methods

### Participants

The pediatric subjects in this study were part of a large study on characterizing brain development in normal and high risk children [46]. Written informed consent was obtained from the parents of all participants and the experimental protocols were approved by the University of North Carolina at Chapel Hill review board. We retrospectively identified 147 normal subjects who met the outlined inclusion and exclusion criteria (more details in Text S1 of SI). The participants include 51 neonates (27 male, 23±12 days (SD)); 50 1-year-olds (27 male, 13±1 months) and 46 2-year-olds (28 male, 24±1 months). All images were acquired using a 3T head-only MR scanner (Allegra, Siemens Medical Systems, Erlangen, Germany). A 3D MP-RAGE sequence was used to provide anatomical images to co-register all images among subjects. The imaging parameters were as follows: repetition time (TR) = 1820 ms; echo time (TE) = 4.38 ms; inversion time = 1100 ms; 144 slices; and voxel size  =  1×1×1 mm3. For the resting functional connectivity MRI (rfcMRI) studies, a T2*-weighted EPI sequence was used to acquire images. The imaging parameters were as follows: TR = 2 sec, TE = 32 ms; 33 slices; and voxel size  = 4×4×4 mm3. This sequence was repeated 150 times so as to providing time series images.

### Preprocessing

The preprocessing steps included exclusion of voxels outside of the brain using FSL (FMRIB, Oxford University, U.K.), time shifting, motion correction, spatial smoothing (6-mm full width at half maximum Gaussian kernel), linear trend removal, and low pass filtering (<0.08 Hz). Nuisance signals from ventricles, white-matter, and global signal were regressed out using linear regression [47]. The first 10 time points of the rfcMRI data were excluded to allow magnetization to reach an equilibrium condition. Subsequently, rfcMRI data of the first available time point was co-registered to the corresponding T1-weighted MP-RAGE structural images using rigid body alignment. For within-group registration, longitudinal T1 images from a subject not included in this study and scanned at 3 wk, 1 yr and 2 yr olds were selected as templates for the corresponding age groups and then an intensity-based HAMMER nonlinear registration [48] was performed to wrap each individual subject to its age-matched template space. Registration between each age group template (from the longitudinal data set) and the MNI space was then done using 4D HAMMER registration. The rationale for using a longitudinal dataset as templates was to achieve a higher registration accuracy though the use of 4D HAMMER registration, which takes into account the longitudinal correlation information. Subsequently, the transformation fields from the 4D HAMMER registration were employed to warp the automatic labeling (AAL) template (90 region of interests (ROIs) covering the cerebral cortex), defined by Tzourio-Mazoyer et al [15] using adult sulcal pattern, to each individual age group template space, achieving an age-specific ROI map. The corresponding correlation matrices were derived based on this age-specific map for all three age groups. The justifications for the utilization of this template are discussed below. First, it has been documented that cortical folding starts at late gestational stage and experiences the trunk of its development in the third trimester [49]. At birth, the major sulcal pattern has already been established and appears similar to that of adults [49], [50]. Second, Hill et al [50] have recently documented that the individual variability of folding pattern in term infants is similar to that of adults. Therefore, given the relatively stable folding pattern throughout postnatal brain development, it is highly plausible that the sulcal pattern based AAL template should also be applicable to our study cohort. In fact, the AAL template has been successfully applied in various developmental/aging studies using graph-theory-analysis [2], [6].

The mean time course of each ROI was separately extracted from each individual subject to construct a 90*90 correlation matrix, which was then Fisher-Z transformed and averaged across subjects to compute the mean correlation matrix for each age group. For each connection within each age group, two-way t-test was performed to calculate a p-value indicating the significance level deviating from zero. This p-value was then used as the threshold to obtain binary sparse matrices across the cost range between 1% and 50% (number of existing connections over all possible ones) with a step size of 1%. After thresholding, the network structure representations were based on the actual functional connectivity strengths rather than p-values. For individual subject analysis, similar sparse matrices were constructed across the cost range. However, the thresholding was based on the correlation magnitude given the absence of individual-subject based p-value for each edge.

### Whole Brain Analysis

To delineate the overall developmental trend of the brain network topology, whole brain analysis was conducted. Clustering coefficient (*Cl*) and characteristic path length (*Cp*) were calculated where regional *Cl* is defined as the ratio of number of existing edges over all possible ones within a subgraph 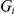
(constituted by the immediate neighbors of node *i*) and whole brain *Cl* was then obtained by averaging over all nodes. *Cp* is defined as the mean shortest path length between all pairs of nodes within the whole graph [7]. By calculating the ratio of clustering coefficient 
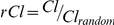
 and characteristic path length 
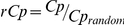
 between the network of interest and a corresponding degree distribution preserved random network, the small-worldness is defined as the ratio of *rCl* and *rCp* with a value above 1 indicating the existence of a small-world topology [7].

To further compare brain wiring efficiencies across the three age groups, local efficiency (LE) and global efficiency (GE) were evaluated based on individual subject's functional network across the cost range between 1% and 50%. LE and GE are measures of information transfer efficiency at local subgraph and whole graph levels, respectively. GE provides more robust measure of global information transfer efficiency than a simple calculation of using path length, especially against those extremely long or infinite (between unconnected nodes) path lengths [14]. The LE and GE were calculated using the equations below.
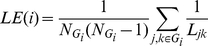
(1)

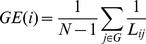
(2)where 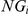 is the number of nodes within a subgraph 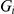 constituted by the immediate neighbors of node *i*. 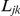 is the minimum path length between each pair of nodes (except the node in question) within the subgraph 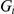. Similarly, *N* is the number of nodes within the whole graph and 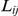 is the minimum path length between the node in question and every other node in the whole graph. The LE and GE of the whole graph were then obtained by averaging across all nodes.

Finally, to test the relationship between efficiency change and anatomical distance, the Euclidian distances between the centers of ROIs were computed. The distances were normalized by the longest connection distance so that different age groups can be compared without the bias of brain size difference. The mean connection distance (labeled as CD) among all non-zero connections within the whole graph at each cost for each subject was calculated and compared across age groups.

### Regional Analysis

Four parameters were compared on a regional level, including LE, GE, degree and nodal maximum connection distance (MD). The definitions of LE and GE were identical as that outlined above with the exception that these values were calculated for each ROI instead of averaging across all ROIs to obtain a global mean. The degree was defined as the number of connections associated with a given node. Finally, MD was obtained as the normalized distance of the longest connection among all connections of a specific node. All regional measures were averaged across the costs of interest and tested between different age groups to delineate significant regional developments.

Besides LE, GE, degree, and MD, another important parameter, betweenness centrality (BC) [19], was similarly evaluated to detect brain functional hubs and assess age-dependent evolution. BC is defined as the fraction of the shortest path between any pair of nodes that travels through the node of interest over all possible ones:
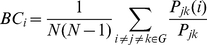
(3)where 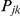 is the total number of shortest paths between nodes *j* and *k* within the whole graph *G* and 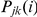 is the total number of shortest paths that pass through node *i*. *N* is the number of nodes within the graph. In all analysis, the calculation of the LE, GE, and BC were done using the online Brain Connectivity Toolbox [51].

### Brain's Resilience to Random and Targeted Attacks

To determine how pediatric brains may sustain disease processes, simulated brain attacks, including random errors and targeted attacks, were conducted. All measures were based on individual subject's functional networks across the cost from 1%∼50%. Random error was simulated by randomly eliminating one node together with all its connections and the interested topological measures of the whole network were subsequently recalculated (repeated 100 times). This process was repeated until 90% of all nodes were eliminated. In contrast, instead of randomly eliminating nodes, attacks specifically targeting the hubs were also simulated. The order of node removal was done in a descending order based on the BC measures. As a reference, the resilience of the pediatric brain networks was compared to that of the random and scale-free networks. The construction of a random network at different costs was done using the Brain Connectivity Toolbox [51] and the construction of a scale-free network was done using the free online code on Matlab Central (http://www.mathworks.com/matlabcentral/fileexchange/11947) based on the Barabasi-Albert algorithm [8].

To demonstrate the behaviors of GE and LE in response to a given removal ratio (either random errors or targeted attacks) and compare across different age groups, the calculated GE and LE for each age group as well as for random and scale-free networks were normalized to the corresponding values obtained with an intact network at a given cost, respectively. This process was repeated for costs ranging between 1% and 50%.

### Statistical Comparison

For the whole brain analysis (including LE, GE, CD, and resilience parameters), the comparison was conducted at each individual cost (from 1%∼50%) and significance was defined as p<0.05 after FDR correction [52]. For connection strength comparison, all connections were compared individually and significant changes were defined as p<0.05 after FDR correction [52]. For the regional analysis (LE, GE, BC, MD, and degree), measurements of the individual nodes were averaged across the costs of interest and compared across groups. Significance was defined as p<0.05 (uncorrected). All tests were conducted using non-parametric kruskal-wallis test.

## Supporting Information

Text S1 Participants.
(DOC)

Figure S1 Anatomical distribution of connections in relation to the age-dependent connectivity strength changes. Red: increasing connections as age grows; Blue: decreasing connections as age grows.
(TIF)

Figure S2 Assessment of changes of brain's network topology using a fixed p-value threshold (p<0.05, FDR corrected). As opposed to Fig. 2 shown in the main text where we fixed the cost so that different age groups have the same number of connections, the results in this figure were obtained by fixing the p-value threshold (p<0.05, FDR corrected) and allowing the cost to vary. (a) the thresholded connectivity matrices for neonates, 1 yr and 2 yr olds; (b) the spring-embedding plots visualizing the corresponding network topology for the three age groups; (c) comparison of local efficiency (LE) and global efficiency (GE) between different age groups. Note here the comparison is based on the 90-ROI nodal efficiency values.
(TIF)

Figure S3 Assessment of the changes of brain's network topology using a fixed connection strength threshold (r>0.2). As opposed to Fig. 2 shown in the main text where we fixed the cost so that different age groups have the same number of connections for comparison, the results in this figure are obtained by fixing the r-value threshold (r>0.2) and allowing the cost to vary. (a) the thresholded connectivity matrices for neonates, 1 yr and 2 yr olds; (b) the spring-embedding plots visualizing the corresponding network topology for the three age groups; (c) bar plot of the corresponding connection density in three age groups; (d) the histogram of the normalized anatomical distance associated with the reserved connections in each age group's network; (e) comparison of local efficiency (LE) and global efficiency (GE) between different age groups. Note here the comparison is based on the 90-ROI nodal efficiency values.
(TIF)

Figure S4 Regional developments of LE (a), GE (b), nodal maximum connection distance (MD) (c) and Degree (d), measured at cost 10%. Both regional increase (represented by yellow to red colors) and decrease (represented by green to blue colors) are shown on the same brain surface. The left column shows the changes from neonates to 1 yr olds while the right column shows the changes from 1 yr to 2 yr olds. Comparing with the results obtained by averaging the cost between 4%∼21%, 17 out of 22 (77.3%) regions showing significant changes of LE at cost 10% from neonates to 1 yr olds also show significant changes when measured by averaging between 4%∼21%; from 1 yr to 2 yr olds, the ratio is 3 out 5 (60.0%); For GE, the corresponding ratio is 44 out of 47 (93.6%) from neonates to 1 yr olds and 8 out 9 (88.9%) from 1 yr to 2 yr olds; For MD, the ratio is 39 out 45 (86.7%) from neonates to 1 yr olds and 11 out of 23 (47.8%) from 1 yr to 2 yr olds; Finally for Degree, the ratio is 23 out of 25 (92.0%) from neonates to 1 yr olds and 8 out of 9 (88.9%) from 1 yr to 2 yr olds.
(TIF)

Figure S5 Regional developments of LE (**a**), GE (**b**), nodal maximum connection distance (MD) (**c**) and Degree (**d**), measured at cost 15%. Both regional increase (represented by yellow to red colors) and decrease (represented by green to blue colors) are shown on the same brain surface. The left column shows the changes from neonates to 1 yr olds while the right column shows the changes from 1 yr to 2 yr olds. Comparing with the results obtained by averaging the cost between 4%∼21%, 21 out of 30 (70.0%) regions showing significant changes of LE at cost 10% from neonates to 1 yr olds also show significant changes when measured by averaging between 4%∼21%; from 1 yr to 2 yr olds, the ratio is 3 out 7 (42.9%); For GE, the corresponding ratio is 40 out of 41 (97.6%) from neonates to 1 yr olds and 8 out 10 (80.0%) from 1 yr to 2 yr olds; For MD, the ratio is 37 out 42 (88.1%) from neonates to 1 yr olds and 10 out of 24 (41.7%) from 1 yr to 2 yr olds; Finally for Degree, the ratio is 21 out of 28 (75.0%) from neonates to 1 yr olds and 6 out of 9 (66.7%) from 1 yr to 2 yr olds.
(TIF)

Figure S6 The list of regional local efficiency (LE) measures across the whole brain. Bar plots represent the mean values across different subjects within each age group and whiskers represent the standard deviations. In addition, the mean value of each region is also visualized on brain surface shown above each bar plot to better delineate the spatial distribution pattern.
(TIF)

Figure S7 The list of regional global efficiency (GE) measures across the whole brain. Bar plots represent the mean values across different subjects within each age group and whiskers represent the standard deviations. In addition, the mean value of each region is also visualized on brain surface shown above each bar plot to better delineate the spatial distribution pattern.
(TIF)

Figure S8 The list of regional maximum distance (MD) measures across the whole brain. Bar plots represent the mean values across different subjects within each age group and whiskers represent the standard deviations. In addition, the mean value of each region is also visualized on brain surface shown above each bar plot to better delineate the spatial distribution pattern.
(TIF)

Figure S9 The list of regional degree measures across the whole brain. Bar plots represent the mean values across different subjects within each age group and whiskers represent the standard deviations. In addition, the mean value of each region is also visualized on brain surface shown above each bar plot to better delineate the spatial distribution pattern.
(TIF)

Figure S10 The list of regional betweenness centrality (BC) measures across the whole brain. Bar plots represent the mean values across different subjects within each age group and whiskers represent the standard deviations. The top 10 hubs are highlighted in red. In addition, the mean value of each region is also visualized on brain surface shown above each bar plot to better delineate the spatial distribution pattern.
(TIF)

Figure S11 The global efficiency (GE) curves in response to simulated random errors and targeted attacks across the cost range of 10%∼50% (at a step size of 10%) for all three age groups. Consistently across all costs, the three pediatric groups show similar resilience to random errors as that of a random network but considerably higher resilience to targeted attacks than that of a scale-free network. The age-dependent improvement of resilience in response to random errors seems to be minimal, but a dramatic improvement of resilience to targeted attack is observed from neonates to 1 yr olds.
(TIF)

Figure S12 The local efficiency (LE) curves in response to simulated random errors and targeted attacks across the cost range of 10%∼50% (at a step size of 10%) for all three age groups. The LE curves for all three age groups are generally higher than both the random and scale-free networks across most of the cost range although the random network becomes more comparable with the study cohorts at costs >40% under targeted attacks. In terms of age-dependent development, the improvement in resilience to random errors is minimal but is highly dramatic between neonates and 1 yr olds under targeted attacks.
(TIF)

Figure S13 Comparison of brain's resilience to random errors and targeted attacks with synthetic small-world networks constructed using the Watts&Strogatz's model. Ten synthetic networks (90 nodes, cost 10%) with rewiring probability from 0.1 to 1 at a step size of 0.1 were constructed and different resilience measures were calculated. The resilience curves of the synthetic small-world networks are shown in dashed black lines with increasing line width from that of rewiring probability of 0.1 to 1. The corresponding curves of pediatric groups are shown in colored lines as labeled.
(TIF)

Table S1 List of regions in anatomical sub-divisions.
(DOCX)

Table S2 Abbreviations of brain regions.
(DOCX)

Table S3 Regional Development of Local Efficiency (LE).
(DOCX)

Table S4 Regional Development of Global Efficiency (GE).
(DOCX)

Table S5 Regional Development of Maximum Connection Distance (MD).
(DOCX)

Table S6 Regional Development of Degree.
(DOCX)
