# Supplementary material for: Temporal and Spatial Evolution of Brain Network Topology during the First Two Years of Life
Source: PLoS One. 2011 Sep 23;6(9):e25278. doi: 10.1371/journal.pone.0025278 (PMC3179501; doi:10.1371/journal.pone.0025278)
Supplement: Text S1 — Participants. (DOC) [file pone.0025278.s001.doc]

**Supporting Online Materials**

**Text S1**

**Participants**

The pediatric subjects in this study were part of a large study on characterizing brain development in normal and high risk children [1]. The inclusion criteria were birth between the gestational ages of 35 and 42 weeks, weight that was appropriate for the gestational age, and the absence of major pregnancy and delivery complications as defined in the exclusion criteria. Exclusion criteria included maternal pre-eclampsia, placental abruption, neonatal hypoxia, any neonatal illness requiring greater than 1 day NICU stay, mother with HIV, any mother actively using illegal drugs/narcotics during pregnancy, any chromosomal or major congenital abnormality. Informed consent was obtained from the parents and the experimental protocols were approved by the institutional review board. None of the subjects were sedated for MR imaging. Before imaging, the children were fed, swaddled, and fitted with ear protection. All subjects slept during the imaging examination.

In addition to the above outlined exclusion criteria, subjects experiencing excessive head motion during scan were also excluded. This was accomplished through carefully pre-screening raw MRI images by evaluating MR signal patterns at a number of pixel locations for each subject. When abrupt signal changes and/or spike-like signal were observed in the measured BOLD signal, the subjects were excluded from data analysis. Therefore, only subjects exhibited no apparent signal discontinuity were included in this study. To further ensure that our study cohorts represent normal and healthy subjects, a board-certified neuroradiologist (JKS) reviewed all images to verify that there were no apparent abnormalities in the acquired MR images. We thus retrospectively identified 147 normal subjects who met the above outlined inclusion and exclusion criteria. The participants include 51 neonates (27M, 23$\pm$12days (SD)); 50 1-year-olds (27M, 13$\pm$1mons) and 46 2-year-olds (28M, 24$\pm$1mons).

**References**

1. Gilmore JH, Lin W, Prastawa MW, Looney CB, Vetsa YS, et al. (2007) Regional gray matter growth, sexual dimorphism, and cerebral asymmetry in the neonatal brain. J Neurosci 27: 1255-1260.
