# Supplementary figures and images for: Temporal and Spatial Evolution of Brain Network Topology during the First Two Years of Life
Source: PLoS One. 2011 Sep 23;6(9):e25278. doi: 10.1371/journal.pone.0025278 (PMC3179501; doi:10.1371/journal.pone.0025278)

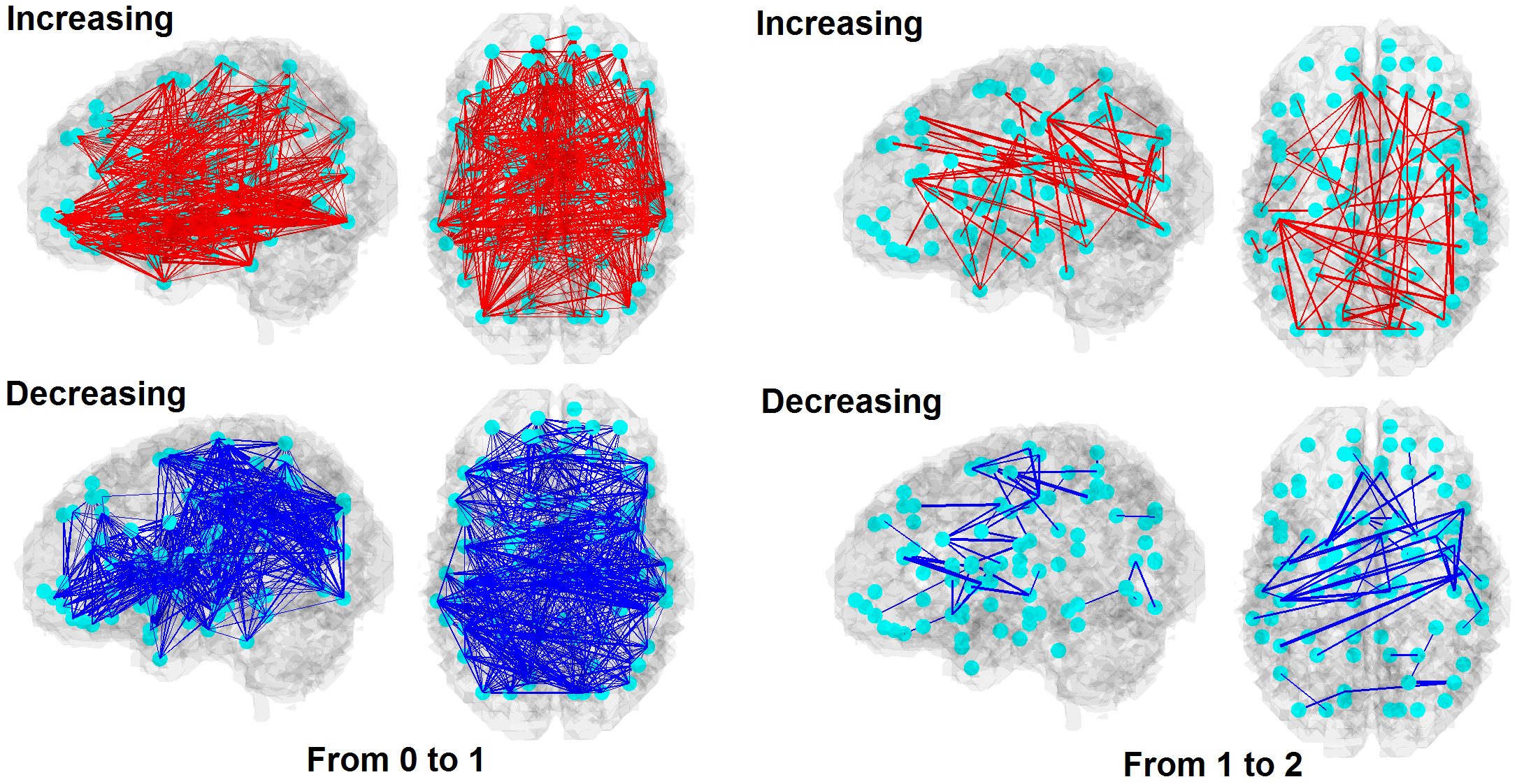

Supplement: Figure S1 — Anatomical distribution of connections in relation to the age-dependent connectivity strength changes. Red: increasing connections as age grows; Blue: decreasing connections as age grows. (TIF) [file pone.0025278.s002.tif]

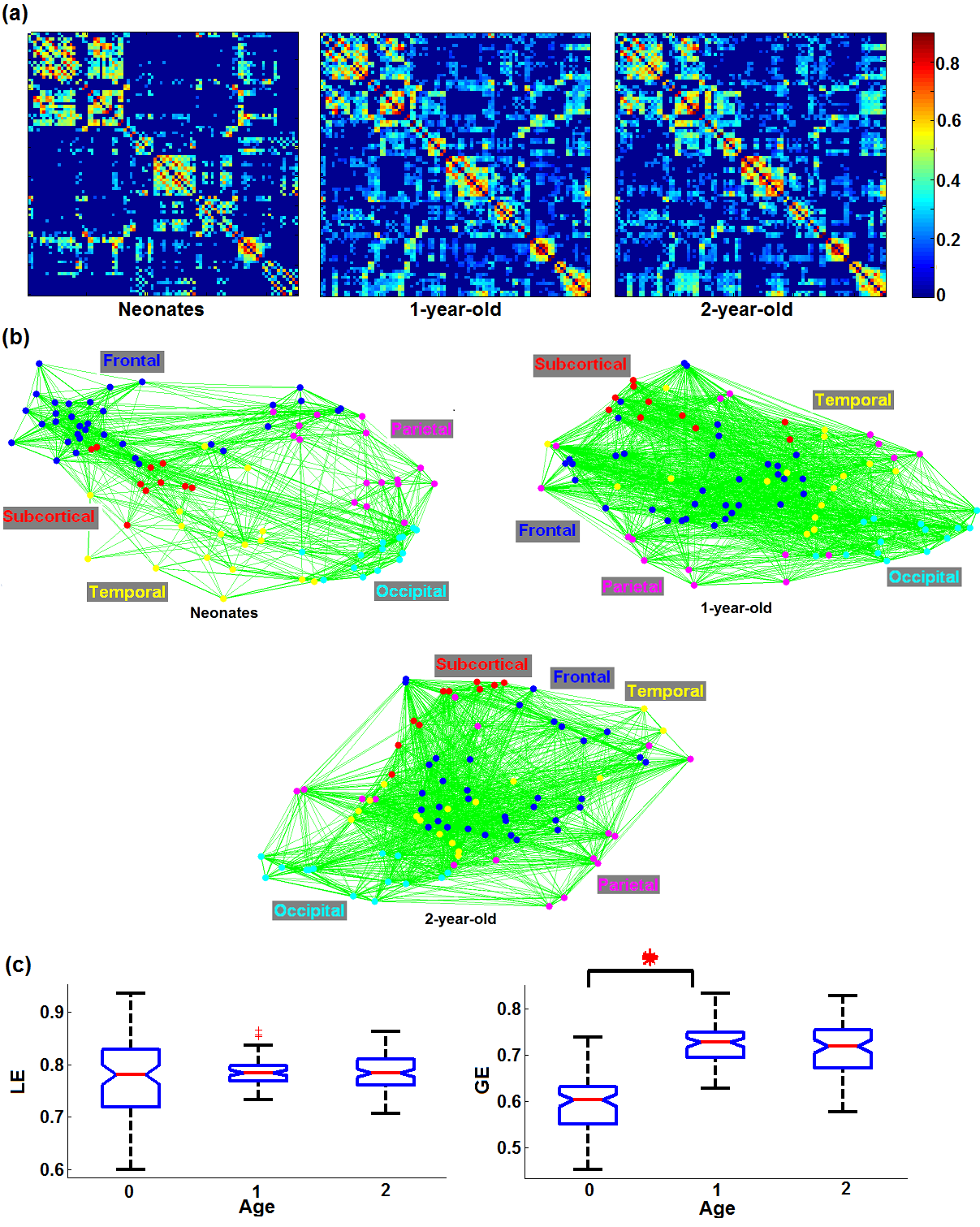

Supplement: Figure S2 — Assessment of changes of brain's network topology using a fixed p-value threshold (p<0.05, FDR corrected). As opposed to Fig. 2 shown in the main text where we fixed the cost so that different age groups have the same number of connections, the results in this figure were obtained by fixing the p-value threshold (p<0.05, FDR corrected) and allowing the cost to vary. (a) the thresholded connectivity matrices for neonates, 1 yr and 2 yr olds; (b) the spring-embedding plots visualizing the corresponding network topology for the three age groups; (c) comparison of local efficiency (LE) and global efficiency (GE) between different age groups. Note here the comparison is based on the 90-ROI nodal efficiency values. (TIF) [file pone.0025278.s003.tif]

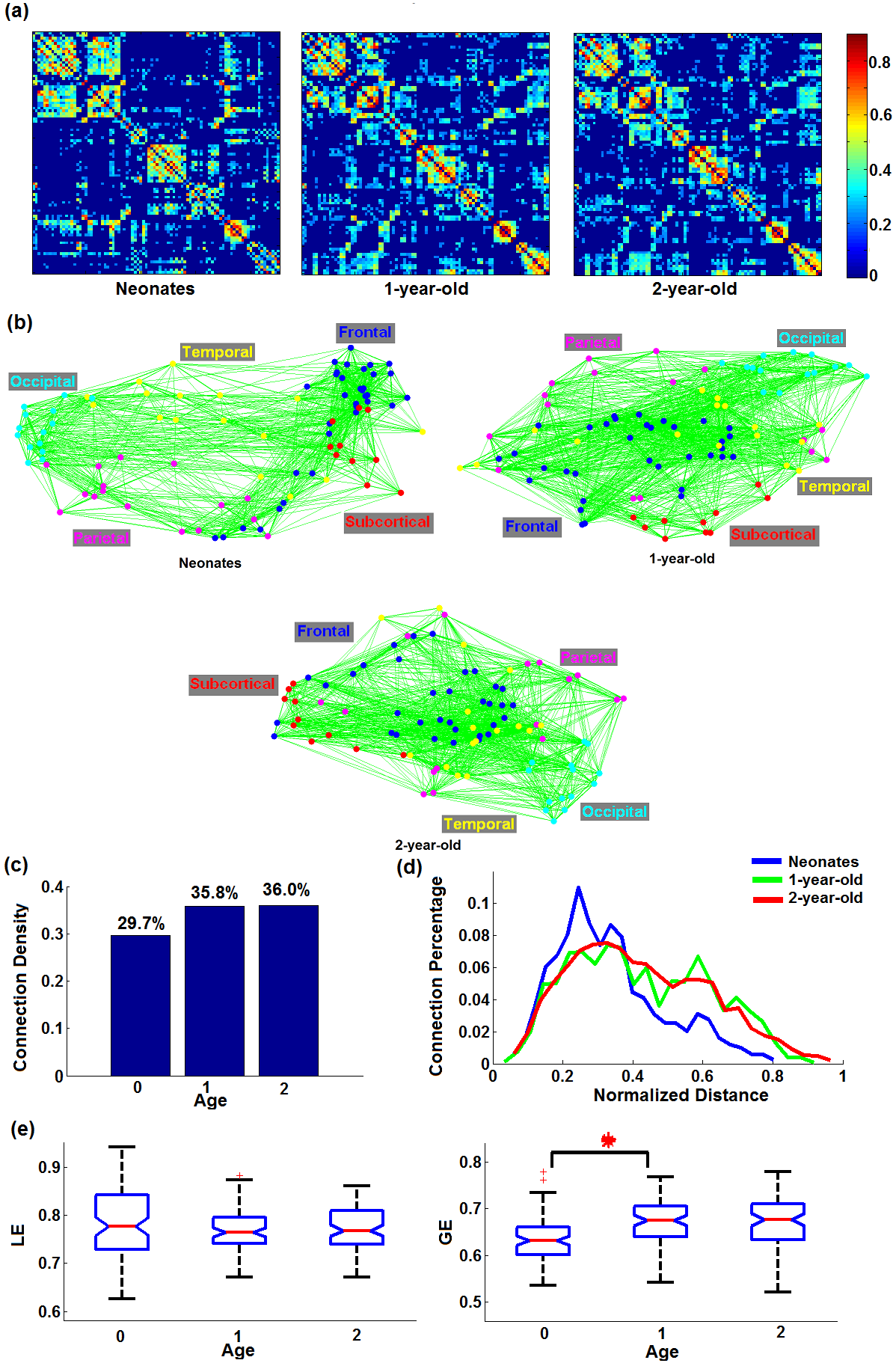

Supplement: Figure S3 — Assessment of the changes of brain's network topology using a fixed connection strength threshold (r>0.2). As opposed to Fig. 2 shown in the main text where we fixed the cost so that different age groups have the same number of connections for comparison, the results in this figure are obtained by fixing the r-value threshold (r>0.2) and allowing the cost to vary. (a) the thresholded connectivity matrices for neonates, 1 yr and 2 yr olds; (b) the spring-embedding plots visualizing the corresponding network topology for the three age groups; (c) bar plot of the corresponding connection density in three age groups; (d) the histogram of the normalized anatomical distance associated with the reserved connections in each age group's network; (e) comparison of local efficiency (LE) and global efficiency (GE) between different age groups. Note here the comparison is based on the 90-ROI nodal efficiency values. (TIF) [file pone.0025278.s004.tif]

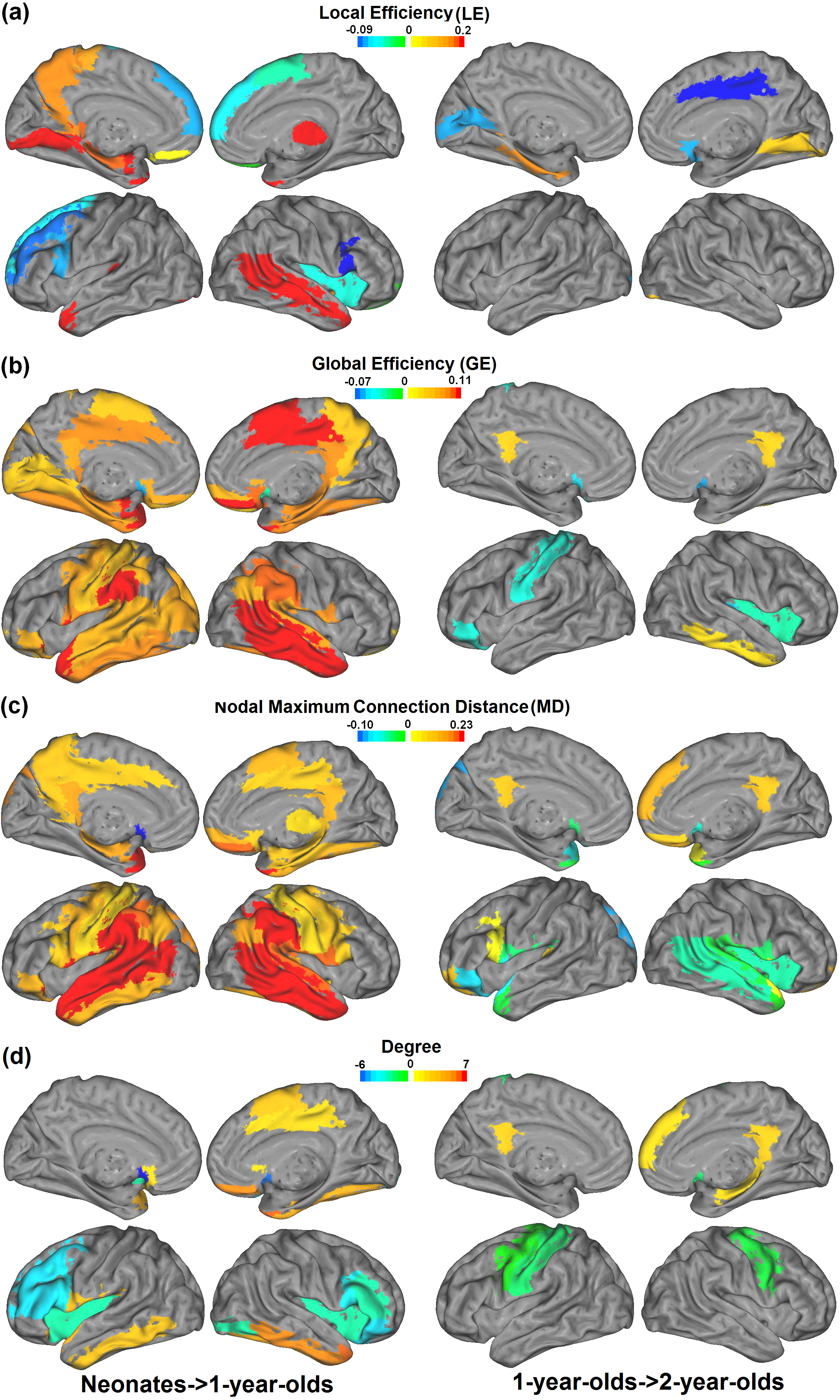

Supplement: Figure S4 — Regional developments of LE (a), GE (b), nodal maximum connection distance (MD) (c) and Degree (d), measured at cost 10%. Both regional increase (represented by yellow to red colors) and decrease (represented by green to blue colors) are shown on the same brain surface. The left column shows the changes from neonates to 1 yr olds while the right column shows the changes from 1 yr to 2 yr olds. Comparing with the results obtained by averaging the cost between 4%∼21%, 17 out of 22 (77.3%) regions showing significant changes of LE at cost 10% from neonates to 1 yr olds also show significant changes when measured by averaging between 4%∼21%; from 1 yr to 2 yr olds, the ratio is 3 out 5 (60.0%); For GE, the corresponding ratio is 44 out of 47 (93.6%) from neonates to 1 yr olds and 8 out 9 (88.9%) from 1 yr to 2 yr olds; For MD, the ratio is 39 out 45 (86.7%) from neonates to 1 yr olds and 11 out of 23 (47.8%) from 1 yr to 2 yr olds; Finally for Degree, the ratio is 23 out of 25 (92.0%) from neonates to 1 yr olds and 8 out of 9 (88.9%) from 1 yr to 2 yr olds. (TIF) [file pone.0025278.s005.tif]

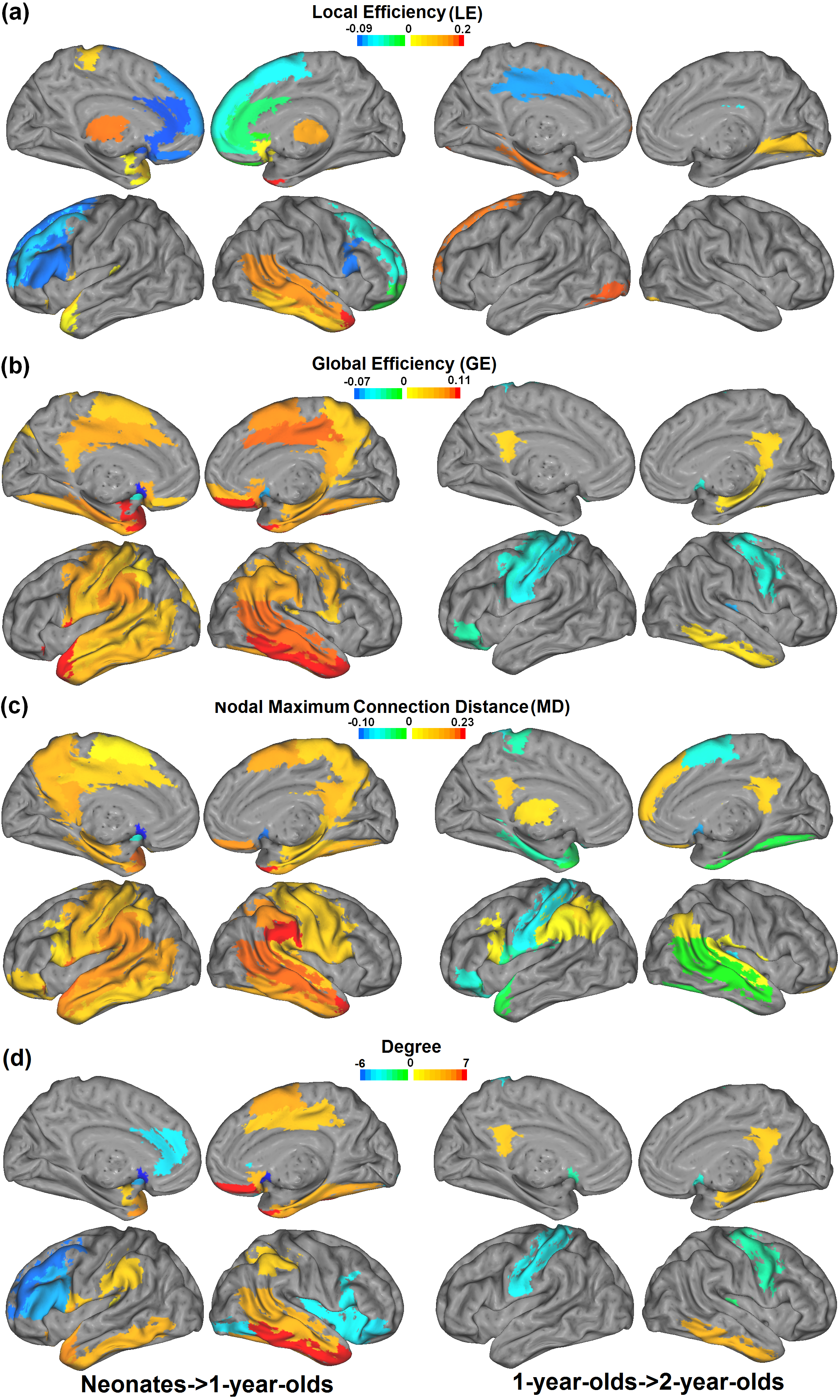

Supplement: Figure S5 — Regional developments of LE (a), GE (b), nodal maximum connection distance (MD) (c) and Degree (d), measured at cost 15%. Both regional increase (represented by yellow to red colors) and decrease (represented by green to blue colors) are shown on the same brain surface. The left column shows the changes from neonates to 1 yr olds while the right column shows the changes from 1 yr to 2 yr olds. Comparing with the results obtained by averaging the cost between 4%∼21%, 21 out of 30 (70.0%) regions showing significant changes of LE at cost 10% from neonates to 1 yr olds also show significant changes when measured by averaging between 4%∼21%; from 1 yr to 2 yr olds, the ratio is 3 out 7 (42.9%); For GE, the corresponding ratio is 40 out of 41 (97.6%) from neonates to 1 yr olds and 8 out 10 (80.0%) from 1 yr to 2 yr olds; For MD, the ratio is 37 out 42 (88.1%) from neonates to 1 yr olds and 10 out of 24 (41.7%) from 1 yr to 2 yr olds; Finally for Degree, the ratio is 21 out of 28 (75.0%) from neonates to 1 yr olds and 6 out of 9 (66.7%) from 1 yr to 2 yr olds. (TIF) [file pone.0025278.s006.tif]

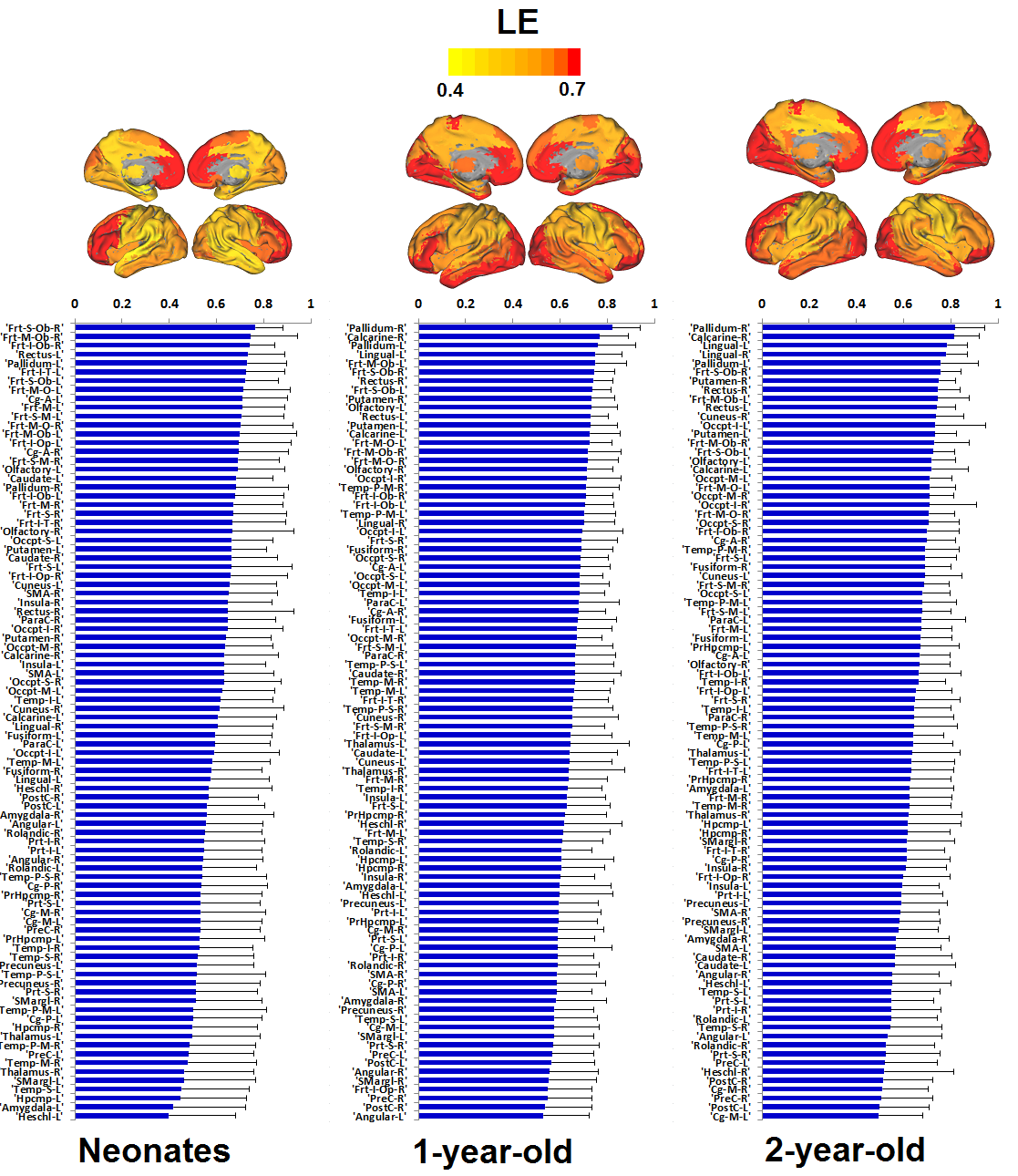

Supplement: Figure S6 — The list of regional local efficiency (LE) measures across the whole brain. Bar plots represent the mean values across different subjects within each age group and whiskers represent the standard deviations. In addition, the mean value of each region is also visualized on brain surface shown above each bar plot to better delineate the spatial distribution pattern. (TIF) [file pone.0025278.s007.tif]

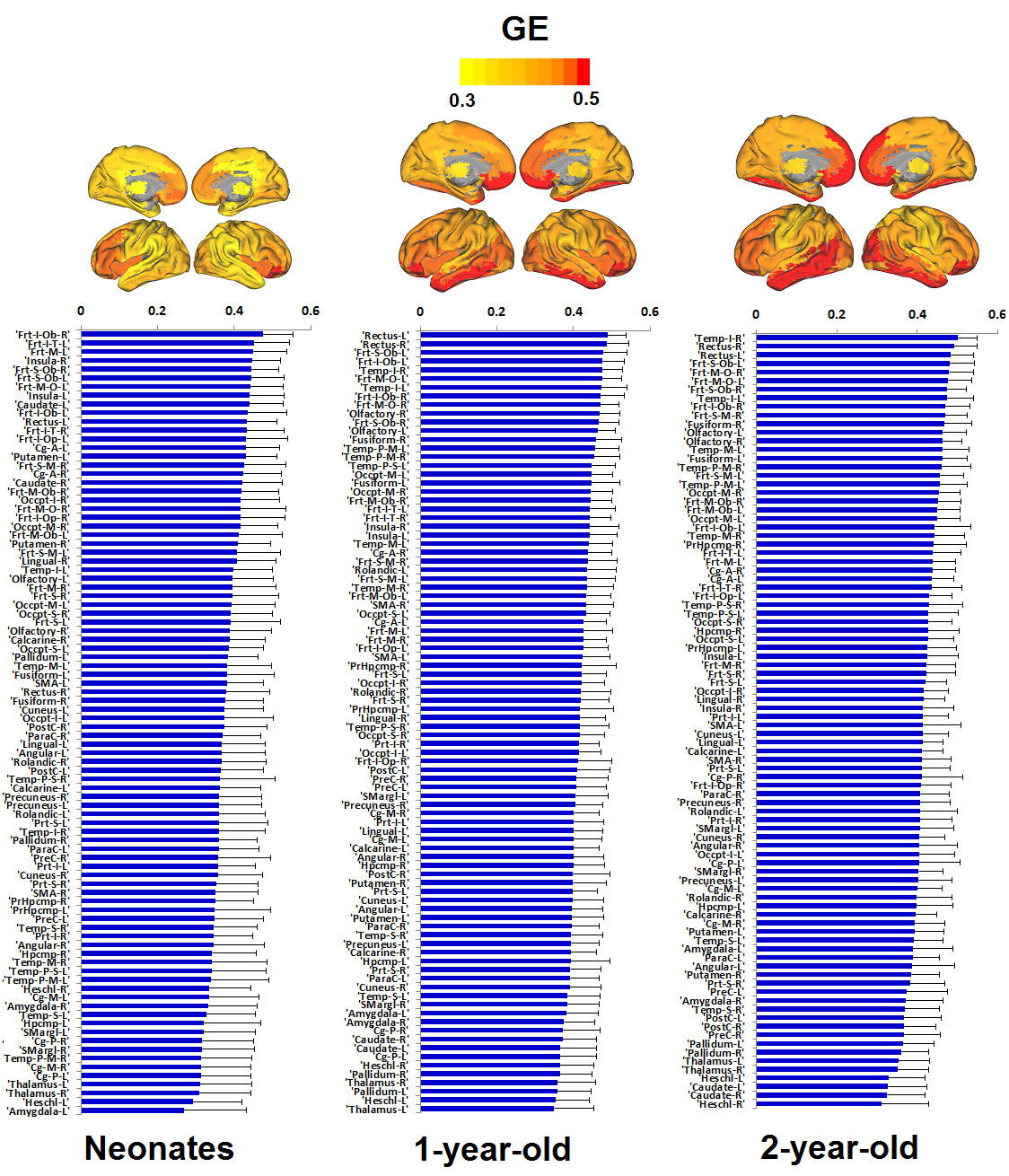

Supplement: Figure S7 — The list of regional global efficiency (GE) measures across the whole brain. Bar plots represent the mean values across different subjects within each age group and whiskers represent the standard deviations. In addition, the mean value of each region is also visualized on brain surface shown above each bar plot to better delineate the spatial distribution pattern. (TIF) [file pone.0025278.s008.tif]

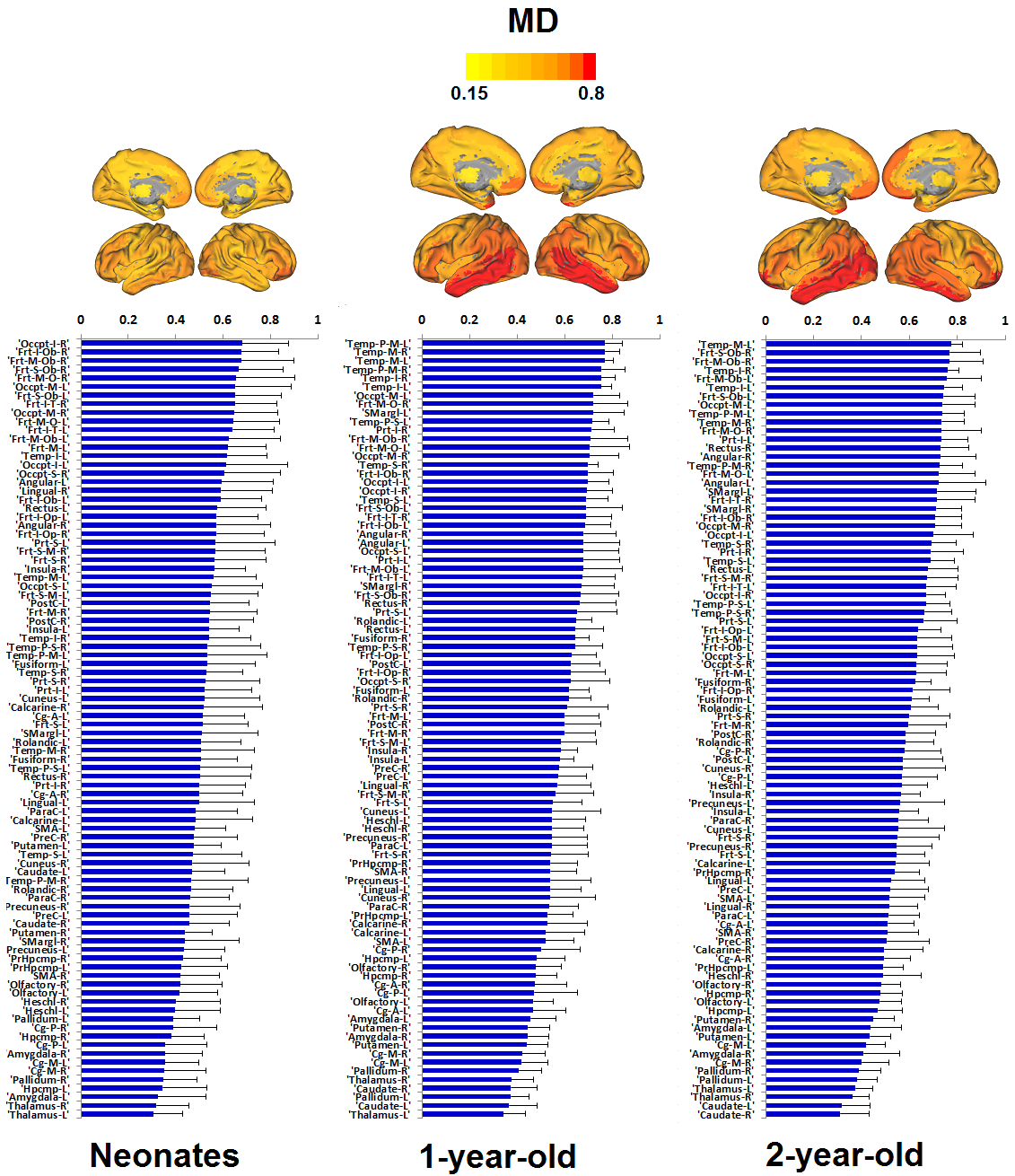

Supplement: Figure S8 — The list of regional maximum distance (MD) measures across the whole brain. Bar plots represent the mean values across different subjects within each age group and whiskers represent the standard deviations. In addition, the mean value of each region is also visualized on brain surface shown above each bar plot to better delineate the spatial distribution pattern. (TIF) [file pone.0025278.s009.tif]

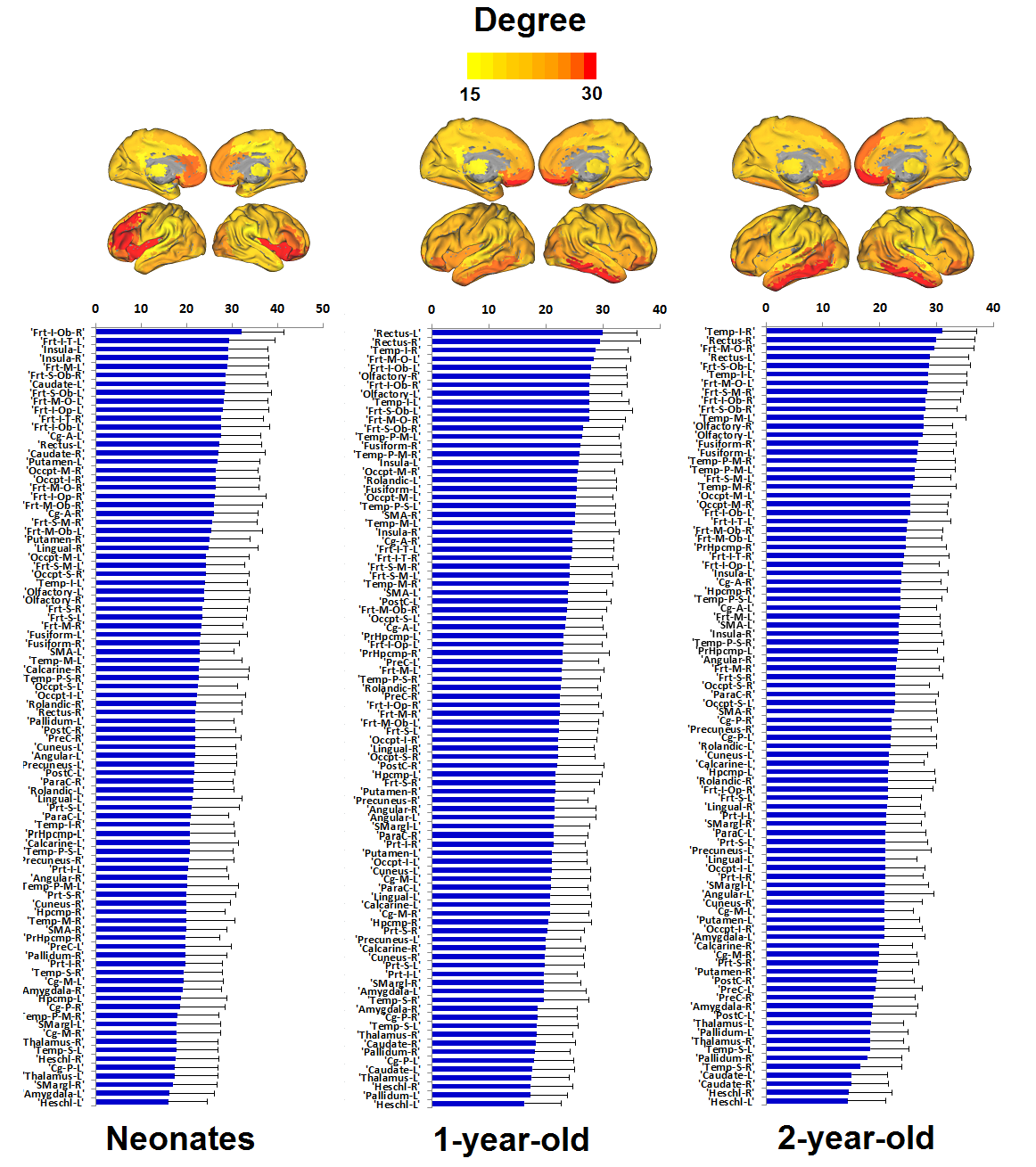

Supplement: Figure S9 — The list of regional degree measures across the whole brain. Bar plots represent the mean values across different subjects within each age group and whiskers represent the standard deviations. In addition, the mean value of each region is also visualized on brain surface shown above each bar plot to better delineate the spatial distribution pattern. (TIF) [file pone.0025278.s010.tif]

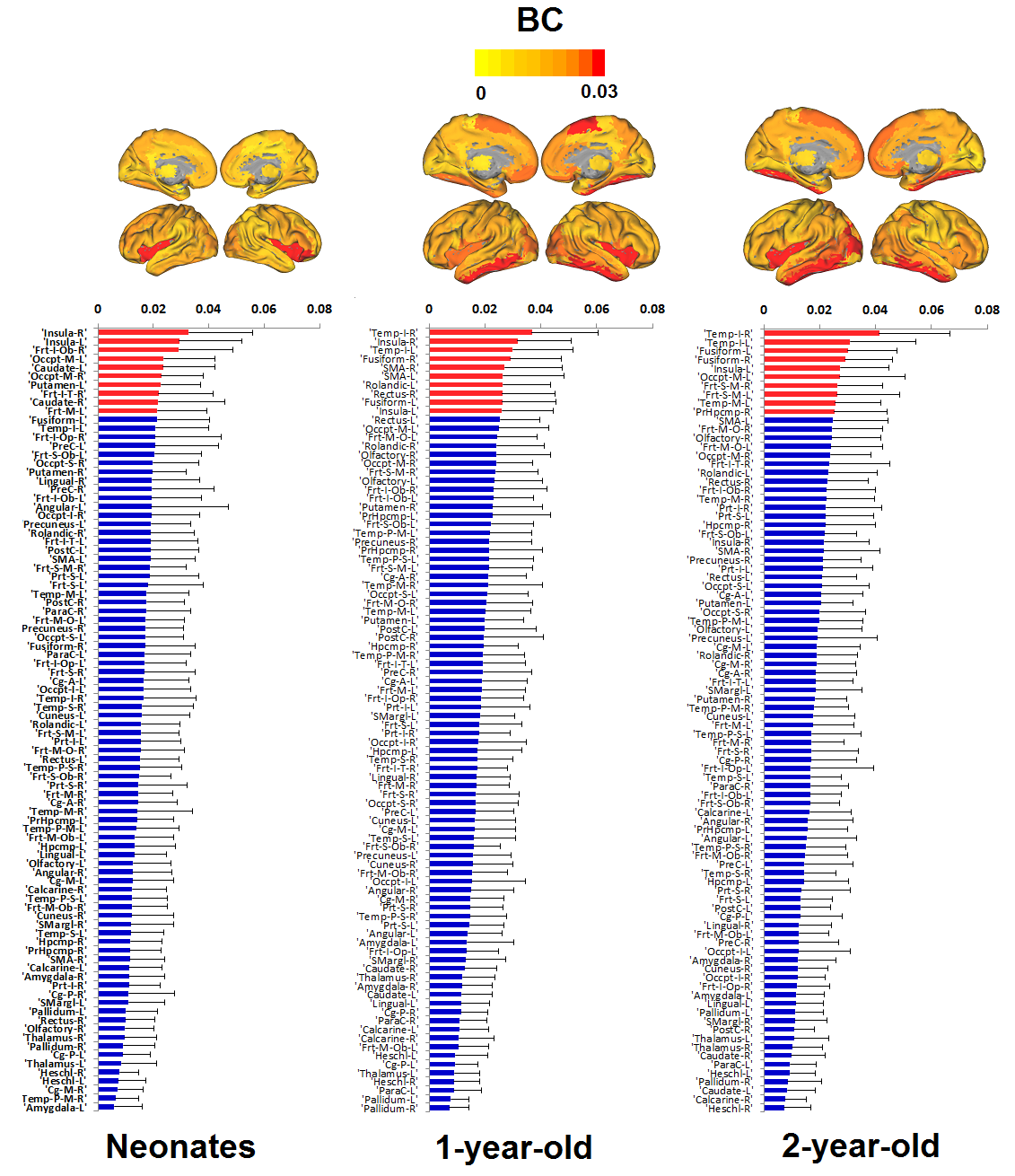

Supplement: Figure S10 — The list of regional betweenness centrality (BC) measures across the whole brain. Bar plots represent the mean values across different subjects within each age group and whiskers represent the standard deviations. The top 10 hubs are highlighted in red. In addition, the mean value of each region is also visualized on brain surface shown above each bar plot to better delineate the spatial distribution pattern. (TIF) [file pone.0025278.s011.tif]

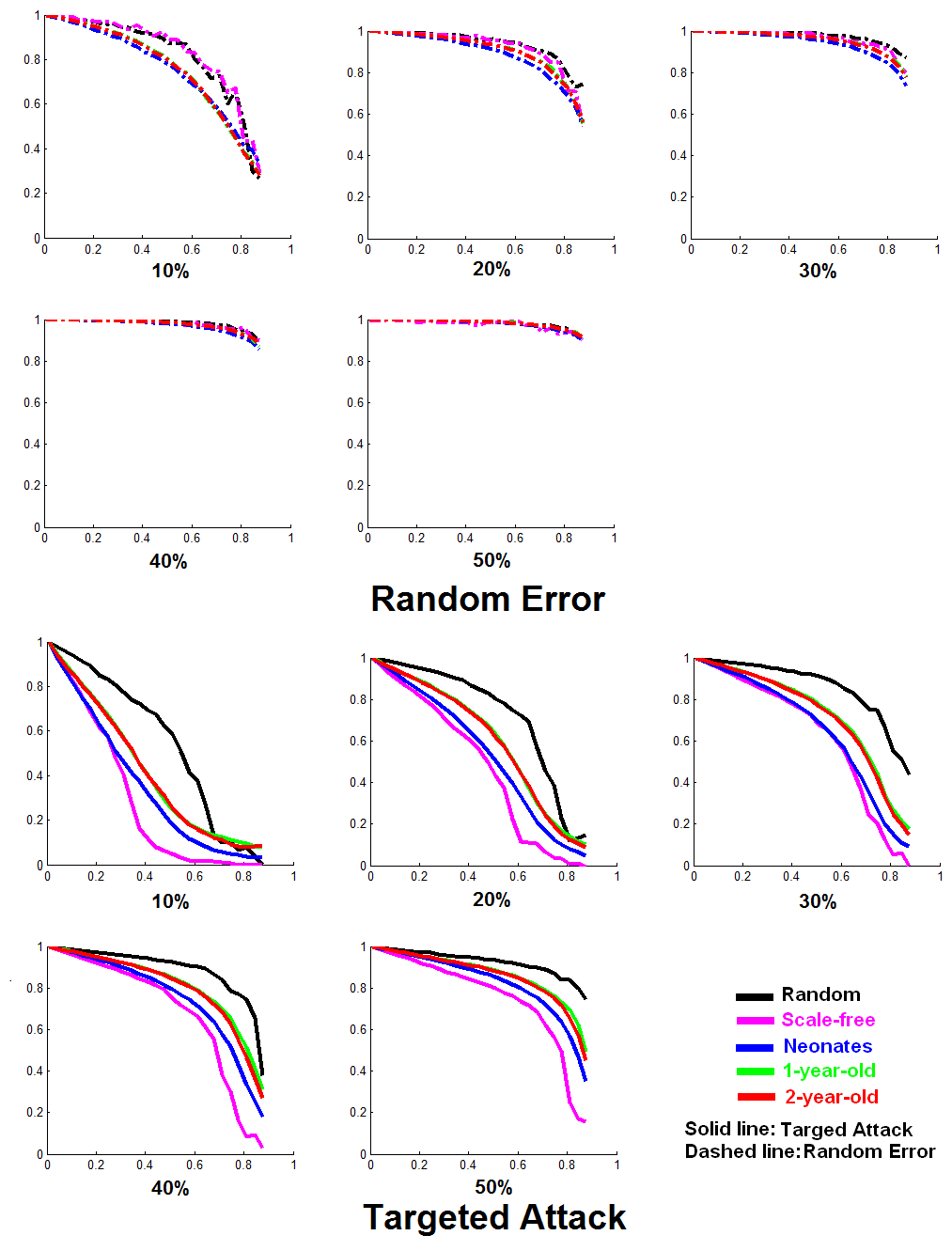

Supplement: Figure S11 — The global efficiency (GE) curves in response to simulated random errors and targeted attacks across the cost range of 10%∼50% (at a step size of 10%) for all three age groups. Consistently across all costs, the three pediatric groups show similar resilience to random errors as that of a random network but considerably higher resilience to targeted attacks than that of a scale-free network. The age-dependent improvement of resilience in response to random errors seems to be minimal, but a dramatic improvement of resilience to targeted attack is observed from neonates to 1 yr olds. (TIF) [file pone.0025278.s012.tif]

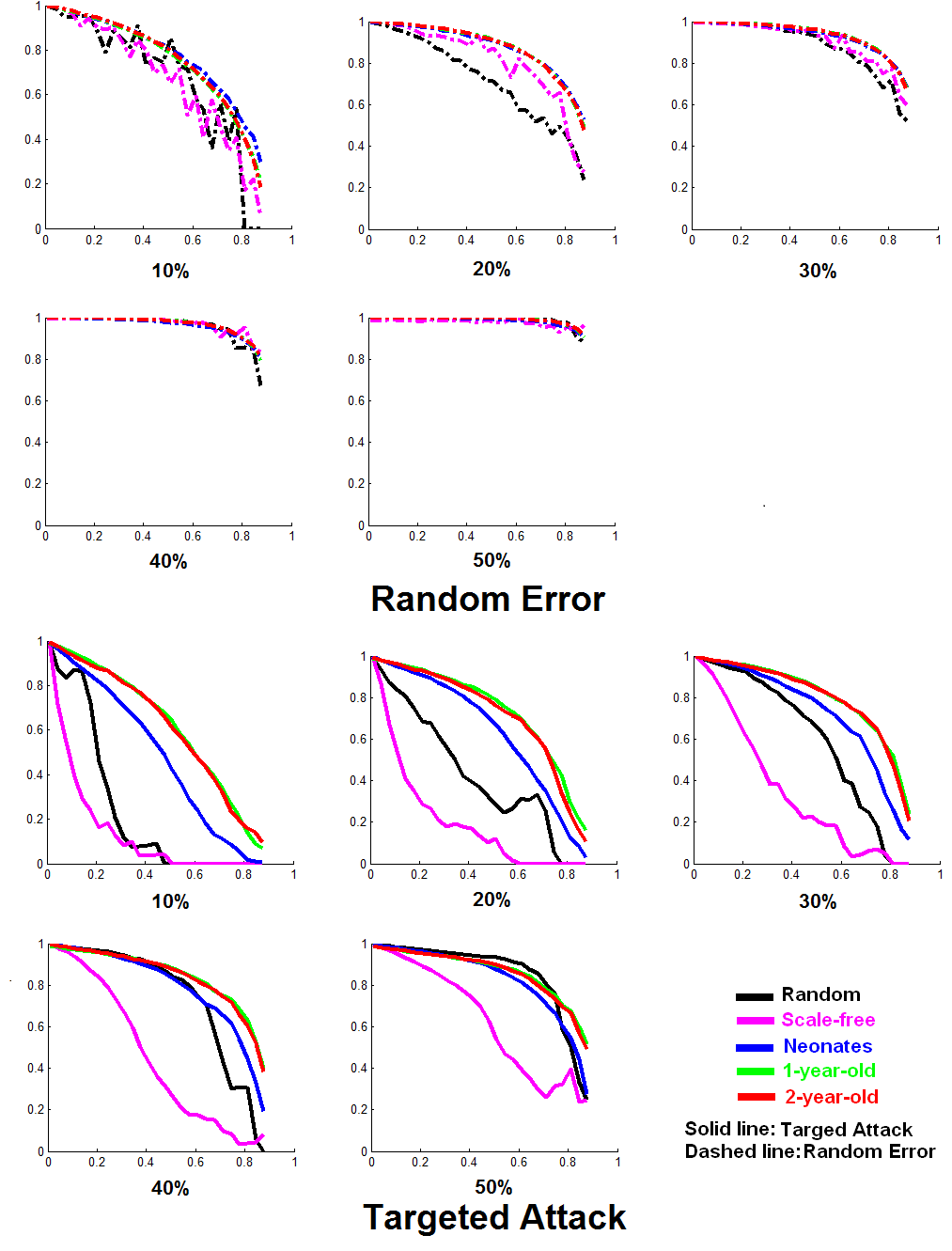

Supplement: Figure S12 — The local efficiency (LE) curves in response to simulated random errors and targeted attacks across the cost range of 10%∼50% (at a step size of 10%) for all three age groups. The LE curves for all three age groups are generally higher than both the random and scale-free networks across most of the cost range although the random network becomes more comparable with the study cohorts at costs >40% under targeted attacks. In terms of age-dependent development, the improvement in resilience to random errors is minimal but is highly dramatic between neonates and 1 yr olds under targeted attacks. (TIF) [file pone.0025278.s013.tif]

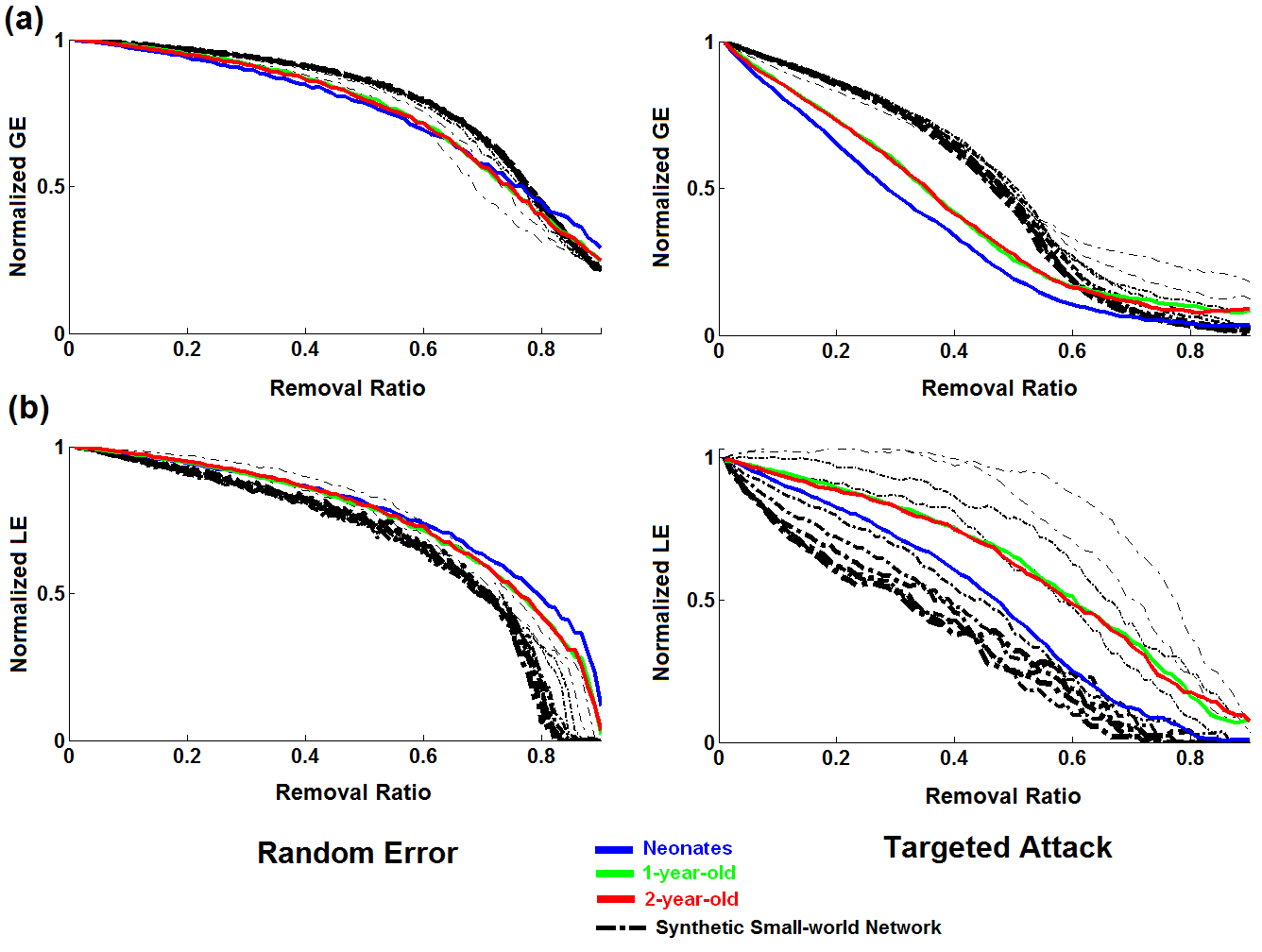

Supplement: Figure S13 — Comparison of brain's resilience to random errors and targeted attacks with synthetic small-world networks constructed using the Watts&Strogatz's model. Ten synthetic networks (90 nodes, cost 10%) with rewiring probability from 0.1 to 1 at a step size of 0.1 were constructed and different resilience measures were calculated. The resilience curves of the synthetic small-world networks are shown in dashed black lines with increasing line width from that of rewiring probability of 0.1 to 1. The corresponding curves of pediatric groups are shown in colored lines as labeled. (TIF) [file pone.0025278.s014.tif]
