# Supplementary material for: Temporal and Spatial Evolution of Brain Network Topology during the First Two Years of Life
Source: PLoS One. 2011 Sep 23;6(9):e25278. doi: 10.1371/journal.pone.0025278 (PMC3179501; doi:10.1371/journal.pone.0025278)
Supplement: Table S1 — List of regions in anatomical sub-divisions. (DOCX) [file pone.0025278.s015.docx]

| **Table S1 List of regions in anatomical sub-divisions** | | | |
| --- | --- | --- | --- |
| **Frontal**  **Parietal** | Superior frontal gyrus  Middle frontal gyrus  Inferior opercular frontal gyrus  Inferior triangular frontal gyrus  Superior medial frontal gyrus  Paracentral Lobule  Superior orbital frontal gyrus  Superior medial orbital frontal gyrus  Middle orbital frontal gyrus  Inferior orbital frontal gyrus  Rectus gyrus  Olfactory gyrus  Precentral gyrus  Supplementary motor area  Rolandic operculum  Anterior cingulate cortex  Superior parietal gyrus  Inferior parietal gyrus  Angular gyrus  Supramarginal gyrus  precuneus  Postcentral gyrus  Median cingulate cortex  Posterior cingulated cortex | **Occipital**  **Temporal**  **Sub-cortical** | Superior occipital gyrus  Middle occipital gyrus  Inferior occipital gyrus  Cuneus  Calcarine cortex  Lingual gyrus  Fusiform gyrus  Superior temporal gyrus  Middle temporal gyrus  Inferior temporal gyrus  Heschl gyrus  Temporal pole: superior temporal gyrus  Temporal pole: middle temporal gyrus  Hippocampus  Parahippocampal gyrus  Amygdala  Caudate nucleus  Putamen  Pallidum  Thalamus  Insula |
|  |  |  |  |
