# Supplementary material for: Temporal and Spatial Evolution of Brain Network Topology during the First Two Years of Life
Source: PLoS One. 2011 Sep 23;6(9):e25278. doi: 10.1371/journal.pone.0025278 (PMC3179501; doi:10.1371/journal.pone.0025278)
Supplement: Table S2 — Abbreviations of brain regions. (DOCX) [file pone.0025278.s016.docx]

| **Table S2 Abbreviations of brain regions** | | | |
| --- | --- | --- | --- |
| **Frt-S** | Superior frontal gyrus | **Temp-S** | Superior temporal gyrus |
| **Frt-M** | Middle frontal gyrus | **Temp-M** | Middle temporal gyrus |
| **Frt-I-Op** | Inferior opercular frontal gyrus | **Temp-I** | Inferior temporal gyrus |
| **Frt-I-T** | Inferior triangular frontal gyrus | **Heschl** | Heschl gyrus |
| **Frt-S-M** | Superior medial frontal gyrus | **Occpt-S** | Superior occipital gyrus |
| **ParaC** | Paracentral lobule | **Occpt-M** | Middle occipital gyrus |
| **Frt-S-Ob** | Superior orbital frontal gyrus | **Occpt-I** | Inferior occipital gyrus |
| **Frt-M-O** | Superior medial orbital frontal gyrus | **Cuneus** | Cuneus |
| **Frt-M-Ob** | Middle orbital frontal gyrus | **Calcarine** | Calcarine cortex |
| **Frt-I-Ob** | Inferior orbital frontal gyrus | **Lingual** | Lingual gyrus |
| **Rectus** | Rectus gyrus | **Fusiform** | Fusiform gyrus |
| **Olfactory** | Olfactory gyrus | **Temp-P-S** | Temporal pole: superior temporal gyrus |
| **Prt-S** | Superior parietal gyrus | **Temp-P-M** | Temporal pole: middle temporal gyrus |
| **Prt-I** | Inferior parietal gyrus | **Cg-A** | Anterior cingulate cortex |
| **Angular** | Angular gyrus | **Cg-M** | Median cingulate cortex |
| **SMargl** | Supramarginal gyrus | **Cg-P** | Posterior cingulated cortex |
| **Precuneus** | precuneus | **Hpcmp** | Hippocampus |
| **PreC** | Precentral gyrus | **ParaHpcmp** | Parahippocampal gyrus |
| **PosC** | Postcentral gyrus | **Amygdala** | Amygdala |
| **SMA** | Supplementary motor area | **Caudate** | Caudate nucleus |
| **Rolandic** | Rolandic operculum | **Putamen** | Putamen |
| **Pallidum** | Pallidum | **Thalamus** | Thalamus |
|  |  | **Insula** | Insula |
|  |  |  |  |
