# Supplementary material for: Temporal and Spatial Evolution of Brain Network Topology during the First Two Years of Life
Source: PLoS One. 2011 Sep 23;6(9):e25278. doi: 10.1371/journal.pone.0025278 (PMC3179501; doi:10.1371/journal.pone.0025278)
Supplement: Table S3 — Regional Development of Local Efficiency (LE). (DOCX) [file pone.0025278.s017.docx]

| **Table S3 Regional Development of Local Efficiency (LE)** | | |
| --- | --- | --- |
|  | **From neonates to 1yr olds** | **From 1yr to 2yr olds** |
| **Increase** | \| Hpcmp-L \| Pallidum-R \| \| --- \| --- \| \| Amygdala-L \| Thalamus-R \| \| Calcarine-R \| Thalamus-L \| \| Calcarine-L \| Heschl-L \| \| Lingual-L \| **Temp-P-S-L** \| \| **Fusiform-R** \| **Temp-M-R** \| \| ParaC-L \| **Temp-P-M-R** \| \| Putamen-R \| **Temp-P-M-L** \| \| Putamen-L \| **Temp-I-R** \| | \| ParaHpcmp-L \| \| --- \| \| Lingual-R \| \| Occpt-M-R \| |
| **Decrease** | \| Frt-M-R \| **SMA-R** \| \| --- \| --- \| \| **Frt-M-L** \| SMA-L \| \| Frt-M-Ob-R \| Frt-S-M-R \| \| **Frt-I-Op-R** \| **Insula-R** \| \| **Frt-I-Op-L** \| **Cg-A-L** \| \| **Frt-I-T-L** \|  \| | \| Olfactory-R \| \| --- \| \| Cg-M-L \| \| PosC-L \| \| Caudate-R \| |

Brain regions highlighted in green indicate regional changes of LE accompanied by similar changes (either increase or decrease) in degree as shown in Table. S6.
