# Supplementary material for: Temporal and Spatial Evolution of Brain Network Topology during the First Two Years of Life
Source: PLoS One. 2011 Sep 23;6(9):e25278. doi: 10.1371/journal.pone.0025278 (PMC3179501; doi:10.1371/journal.pone.0025278)
Supplement: Table S4 — Regional Development of Global Efficiency (GE). (DOCX) [file pone.0025278.s018.docx]

| **Table S4 Regional Development of Global Efficiency (GE)** | | |
| --- | --- | --- |
|  | **From neonates to 1yr olds** | **From 1yr to 2yr olds** |
| **Increase** | \| PreC-L \| Occpt-M-L \| \| --- \| --- \| \| Frt-S-Ob-L \| Fusiform-R \| \| Frt-I-Ob-L \| Fusiform-L \| \| Rolandic-R \| PosC-L \| \| Rolandic-L \| Prt-I-R \| \| **SMA-R** \| Prt-I-L \| \| SMA-L \| SMargl-R \| \| Olfactory-R \| SMargl-L \| \| Olfactory-L \| Angular-R \| \| Frt-M-Ob-R \| Precuneus-R \| \| Rectus-R \| Heschl-L \| \| Rectus-L \| Temp-S-R \| \| Cg-M-R \| Temp-S-L \| \| Cg-M-L \| Temp-P-S-L \| \| Cg-P-R \| Temp-M-R \| \| Cg-P-L \| Temp-M-L \| \| Hpcmp-R \| Temp-P-M-R \| \| Hpcmp-L \| Temp-P-M-L \| \| ParaHpcmp-R \| Temp-I-R \| \| ParaHpcmp-L \| Temp-I-L \| \| Amygdala-L \|  \| \| Occpt-S-L \|  \| | \| **Cg-P-R** \| \| --- \| \| **Cg-P-L** \| \| Temp-I-R \| |
| **Decrease** | \| **Caudate-R** \| \| --- \| \| **Caudate-L** \| \| Putamen-L \| | \| **PreC-R** \| \| --- \| \| Frt-I-Ob-L \| \| **PosC-R** \| \| **PosC-L** \| \| **Caudate-R** \| \| **Caudate-L** \| \| Heschl-R \| |

The brain regions highlighted in red indicate a concurrent increase of GE/MD/degree while brain regions with concurrent decreases of GE/MD/degree are marked in blue.
