# Supplementary material for: Temporal and Spatial Evolution of Brain Network Topology during the First Two Years of Life
Source: PLoS One. 2011 Sep 23;6(9):e25278. doi: 10.1371/journal.pone.0025278 (PMC3179501; doi:10.1371/journal.pone.0025278)
Supplement: Table S5 — Regional Development of Maximum Connection Distance (MD). (DOCX) [file pone.0025278.s019.docx]

| **Table S5 Regional Development of Maximum Connection Distance (MD)** | | |
| --- | --- | --- |
|  | **From neonates to 1yr olds** | **From 1yr to 2yr olds** |
| **Increase** | \| PreC-R \| Prt-I-L \| \| --- \| --- \| \| PreC-L \| SMargl-R \| \| Frt-I-Ob-L \| SMargl-L \| \| Rolandic-R \| Angular-R \| \| Rolandic-L \| Precuneus-R \| \| **SMA-R** \| Precuneus-L \| \| Rectus-R \| ParaC-R \| \| Cg-M-R \| Pallidum-R \| \| Cg-M-L \| Thalamus-R \| \| Cg-P-R \| Heschl-R \| \| Cg-P-L \| Heschl-L \| \| Hpcmp-R \| Temp-S-R \| \| Hpcmp-L \| Temp-S-L \| \| ParaHpcmp-R \| Temp-P-S-R \| \| ParaHpcmp-L \| Temp-P-S-L \| \| Amygdala-R \| Temp-M-R \| \| Amygdala-L \| Temp-M-L \| \| Occpt-S-L \| Temp-P-M-R \| \| Fusiform-R \| Temp-P-M-L \| \| PosC-L \| Temp-I-R \| \| Prt-I-R \| Temp-I-L \| | \| Frt-S-Ob-R \| \| --- \| \| Frt-M-Ob-L \| \| \| **Frt-S-M-R** \| \| \| **Cg-P-R** \| \| \| **Cg-P-L** \| \| |
| **Decrease** | \| **Caudate-R** \| \| --- \| \| **Caudate-L** \| \| Putamen-L \| | \| **Rolandic-L** \| \| --- \| \| ParaHpcmp-L \| \| Lingual-R \| \| **Caudate-R** \| \| **Caudate-L** \| \| Heschl-R \| \| Temp-S-R \| \| Temp-P-S-L \| \| Temp-M-R \| \| Temp-P-M-R \| |

The brain regions highlighted in red indicate a concurrent increase of GE/MD/degree while brain regions with concurrent decreases of GE/MD/degree are marked in blue.
