# Supplementary material for: Temporal and Spatial Evolution of Brain Network Topology during the First Two Years of Life
Source: PLoS One. 2011 Sep 23;6(9):e25278. doi: 10.1371/journal.pone.0025278 (PMC3179501; doi:10.1371/journal.pone.0025278)
Supplement: Table S6 — Regional Development of Degree. (DOCX) [file pone.0025278.s020.docx]

| **Table S6 Regional Development of Degree** | | |
| --- | --- | --- |
|  | **From neonates to 1yr olds** | **From 1yr to 2yr olds** |
| **Increase** | \| Rolandic-L \| **Temp-P-S-L** \| \| \| --- \| --- \| --- \| \| SMA-R \| \| **Temp-P-M-R** \| \| Olfactory-L \| **Temp-P-M-L** \| \| \| Rectus-R \| **Temp-I-R** \| \| \| Cg-M-R \| Temp-I-L \| \| \| **Fusiform-R** \|  \| \| | \| **Frt-S-M-R** \| \| --- \| \| **Cg-P-R** \| \| **Cg-P-L** \| \| Hpcmp-R \| |
| **Decrease** | \| **Frt-M-L** \| **Cg-A-L** \| \| --- \| --- \| \| **Frt-I-Op-R** \| Occpt-I-R \| \| **Frt-I-Op-L** \| **Caudate-R** \| \| Frt-I-T-R \| **Caudate-L** \| \| **Frt-I-T-L** \| Putamen-L \| \| Frt-I-Ob-R \| Pallidum-L \| \| **Insula-R** \|  \| \| Insula-L \|  \| | \| **PreC-R** \| \| --- \| \| **PreC-L** \| \| **Rolandic-L** \| \| **PosC-L** \| \| **Caudate-R** \| |

Brain regions highlighted in green indicate regional changes of degree accompanied by similar changes (either increase or decrease) in local efficiency as shown in Table. S3. The brain regions highlighted in red indicate a concurrent increase of GE/MD/degree while brain regions with concurrent decreases of GE/MD/degree are marked in blue.
